# Graphene Energy Transfer for Single‐Molecule Biophysics, Biosensing, and Super‐Resolution Microscopy

**DOI:** 10.1002/adma.202101099

**Published:** 2021-05-03

**Authors:** Izabela Kamińska, Johann Bohlen, Renukka Yaadav, Patrick Schüler, Mario Raab, Tim Schröder, Jonas Zähringer, Karolina Zielonka, Stefan Krause, Philip Tinnefeld

**Affiliations:** ^1^ Institute of Physical Chemistry of the Polish Academy of Sciences Kasprzaka 44/52 Warsaw 01‐224 Poland; ^2^ Department of Chemistry and Center for NanoScience Ludwig‐Maximilians‐Universität München Butenandtstr. 5‐13 81377 München Germany

**Keywords:** biophysics, biosensing, DNA origami, Förster‐type resonance energy transfer, graphene, single molecules, super‐resolution

## Abstract

Graphene is considered a game‐changing material, especially for its mechanical and electrical properties. This work exploits that graphene is almost transparent but quenches fluorescence in a range up to ≈40 nm. Graphene as a broadband and unbleachable energy‐transfer acceptor without labeling, is used to precisely determine the height of molecules with respect to graphene, to visualize the dynamics of DNA nanostructures, and to determine the orientation of Förster‐type resonance energy transfer (FRET) pairs. Using DNA origami nanopositioners, biosensing, single‐molecule tracking, and DNA PAINT super‐resolution with <3 nm *z*‐resolution are demonstrated. The range of examples shows the potential of graphene‐on‐glass coverslips as a versatile platform for single‐molecule biophysics, biosensing, and super‐resolution microscopy.

## Graphical abstract

Graphene is a broadband and unbleachable energy‐transfer acceptor with no need for labeling. To demonstrate the capabilities of graphene‐on‐glass coverslips, a variety of demonstration assays are placed on graphene using DNA origami nanopositioners. The results highlight the potential for single‐molecule biophysics, biosensing, and super‐resolution microscopy.

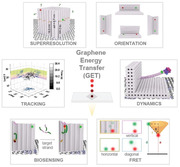

## 1 Introduction

Graphene is the prototypical 2D material whose extraordinary properties including mechanical strength, electrical and thermal conductivity, as well as uniform absorption across the visible spectrum have made it attractive for many research directions.^[^ ^1^, ^2^, ^3^, ^4^, ^5^ ^]^ While only 2.3% of visible light is absorbed, graphene constitutes an efficient acceptor for nonradiative energy transfer for fluorescent dyes in the near‐field in analogy to FRET.^[^ ^6^, ^7^, ^8^, ^9^, ^10^ ^]^ Accordingly, excited state energy is nonradiatively transferred to graphene with a *d* ^−4^ scaling law and a wavelength independent characteristic length scale of ≈18 nm with 50% energy transfer efficiency.^[^ ^6^, ^8^, ^9^ ^]^ The fluorescence quenching property has been used for graphene characterization^[^ ^10^, ^11^, ^12^ ^]^ and for biosensors based on graphene‐related materials such as graphene oxide or reduced graphene oxide^[^ ^13^, ^14^, ^15^, ^16^ ^]^ but only recently its potential for applications in the life science including super‐resolution microscopy has been realized.^[^ ^9^, ^17^, ^18^, ^19^, ^20^ ^]^ Due to graphene energy transfer (GET), the intensity of a fluorescent dye as well as its fluorescence lifetime are reduced as a function of its distance to graphene. This information can be used to determine the position of the dye molecule to graphene and to sensitively report on distance changes. We here introduce graphene‐on‐glass coverslips as a platform for additional functionality and information content in single‐molecule biophysics and biosensing, and to provide 3D information in super‐resolution microscopy and in single‐molecule tracking. To make graphene chemically accessible, we use DNA origami nanostructures as chemical adapters and place demonstration assays at defined distances on top of graphene coverslips.

We exemplify the potential of graphene energy transfer with five different assay formats. First, we show height measurements of dyes based on fluorescence lifetimes. We then sense the orientation of DNA origami nanostructures with four landing surfaces by inserting two fluorescent lifetime reporting dyes. Second, we visualize switching dynamics of a DNA pointer between two binding sites with high time resolution using an autocorrelation scheme that filters for lifetime associated components. This approach also enables detecting the dynamics of a flexible DNA tether influenced by viscosity or target binding. Third, by combining FRET with GET, we determine the orientation of a donor–acceptor pair with respect to the substrate, in both static and dynamic systems. Fourth, we show a biosensing assay with single DNA molecule detection in a novel unquenching assay format that uses graphene as a quencher. Fifth, we combine GET with DNA point accumulation in nanoscale topography (DNA PAINT) super‐resolution imaging and for single‐molecule tracking with resolution below 3 nm in *z* and 6 nm in *x*/*y* on DNA origami structures.

In summary, using graphene energy transfer with graphene‐on‐glass coverslips^[^ ^21^ ^]^ provides many new opportunities for biosensing, single‐molecule biophysics and super‐resolution, ready to be lifted with the aid of DNA origami nanopositioners.

## 2 Results

### 2.1 Distance Determination from Fluorescence Lifetimes

**Figure 1:**
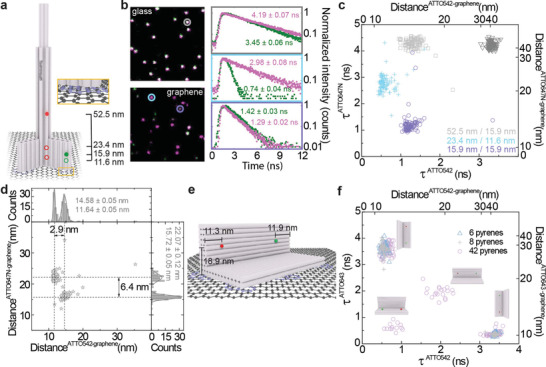
Distance determination from fluorescence lifetimes. a) Sketch of a pillar‐shaped DNA origami structure, with the marked positions of dye molecules, and a zoom‐in of pyrene molecules (orange frame) used for the immobilization of DNA origami structures on graphene. b) Fluorescence intensity maps of DNA origami structure labeled with two dye molecules immobilized on glass (top) and graphene (bottom), together with fluorescence decays and fitted fluorescence lifetime values with standard errors of the fit (right) of a green (green) and a red (magenta) dye marked on the maps: gray—on glass; cyan and violet—on graphene. c) Scatter plot of fluorescence lifetime of colocalized dye molecules (both dye molecules within one DNA origami structure), ATTO542 and ATTO647N, at various heights; each DNA origami sample measured separately on glass (∇) or graphene (**□**, **+**, **○**), with ATTO542/ATTO647N at 15.9/52.5 nm (**□** gray), 11.6/23.4 nm (**+** cyan) or 15.9/15.9 nm (**○** violet) distance from graphene. d) Scatter plot and corresponding histograms with the Gaussian fits of distances from graphene calculated from fluorescence lifetimes (for calculations check Supporting Information) of two mixed DNA origami structures with ATTO542/ATTO647N at 11.6/23.4 and 15.9/15.9 nm distance from graphene. e) Sketch of an L‐shaped DNA origami structure with ATTO643 and ATTO542 positioned at the height of 18.9, and 11.3 or 11.9 nm from the side edges, respectively. f) Scatter plot of fluorescence lifetimes of colocalized dye molecules within L‐shaped DNA origami structure obtained for structures labeled with 6 (**∆** blue), 8 (**+** gray) or 42 (**○** lilac) pyrene molecules, showing four different orientations on graphene (indicated by sketches).

With the known *d*_0_‐value and the *d* ^−4^ distance dependence, the distance of a molecule from graphene can be determined from its fluorescence intensity or analogously, from the fluorescence lifetime as proportional intensive property. Here we determine the distance of molecules from the graphene surface using their fluorescence lifetime. “Single‐molecule grade” graphene‐on‐glass coverslips were prepared by transferring CVD‐grown graphene from copper‐foils to glass coverslips with an optimized protocol that avoids impurities (see Materials and Methods, Supporting Information). We used pyrene‐equipped DNA origami nanopositioners to place fluorescent dyes on top of the graphene coverslips (see **Figure** **1**a). Pyrene modifications ensure a selective orientation of the DNA origami nanopillar on top of graphene.^[^ ^9^ ^]^

Three samples were labeled with a green (ATTO542) and a red (ATTO647N) dye molecule at the heights of 15.9 and 52.5 nm (**1**), 11.6 and 23.4 nm (**2**), 15.9 and 15.9 nm (**3**), respectively.^[^ ^9^ ^]^ Using alternating pulsed laser excitation at 532 and 639 nm, both dyes were measured quasi‐simultaneously by single‐molecule fluorescence lifetime imaging (Figure 1b, see also Supporting Information for sample preparation, selection of dyes, experimental conditions, and data analysis). Single molecules were identified in the fluorescence images and their fluorescence lifetime was determined (example decays are shown in Figure 1b). Reference measurements on glass show a narrow and homogenous population of fluorescence lifetime that is identical for all three samples (Figure 1c, ∇) with an average fluorescence lifetime of 4.3 ± 0.1 ns for ATTO647N and 3.4 ± 0.1 ns for ATTO542. For each nanostructure measured on graphene, shortened fluorescence lifetimes of both dye molecules indicate the proximity of graphene (Figure 1c **□**, **+**, **○**), except for ATTO647N in sample **1**. This one emitter was incorporated at the height of 52.5 nm, which is too far from graphene for measurable shortening of the fluorescence lifetime. In the following experiment, samples **2** and **3** were mixed, immobilized on graphene, and imaged. An example of a fluorescence intensity map and decays obtained for the mixed sample are presented in Figure 1b (lower panel and decays in cyan and violet frames). The scatter plot of the dye–graphene distance (Figure 1d) calculated from the measured values of the fluorescence lifetime of dye molecules (see Figure S1 and calculations, Supporting Information), confirm that the populations of the two DNA origami structures are separated equally well as in the isolated samples (Figure 1c), indicating that differences in *z*‐direction as small as 6.4 and 2.9 nm are easily resolved from the fluorescence lifetimes of single molecules. Next, we used this sensitivity to study the orientation of a DNA origami structure that can bind to graphene in different orientations. The L‐shaped DNA origami structure (Figure 1e) can bind to graphene through pyrene immobilization at the bottom of the structure but also by blunt end stacking to the sides. We placed two dye molecules in the DNA origami structure at the height of 18.9 nm (≈48% and 43% energy transfer efficiency to graphene, for ATTO643 and ATTO542, respectively)^[^ ^9^ ^]^ and 11.3 or 11.9 nm from the side edges, for ATTO643 and ATTO542, respectively, such that the obtained combination of fluorescence lifetime directly reports on the orientation of each individual DNA origami structure. Three tested samples varied by the number of incorporated pyrene molecules. Two samples contained 6 or 8 pyrene molecules and for both, two populations of L‐shaped DNA origami structure positioned on one or the other side were observed (Figure 1f, ∆ and +). Increasing the number of binding strands for pyrene labeled strands to 42 resulted in the appearance of four populations (Figure 1f, **○**) with the expected combination of fluorescence lifetimes showing that binding mediated by pyrenes was adopted by ≈15% of all structures. The population with very short fluorescence lifetimes of both dyes could be explained by binding of the DNA origami structure on the remaining site or by partial degradation of the DNA origami structures. We note that it is advantageous for homogeneous DNA origami nanopositioning on graphene if the *π–π* stacking interactions of both pyrene molecules and DNA bases with graphene are additive and not competitive.

### 2.2 Dynamics with GET

**Figure 2:**
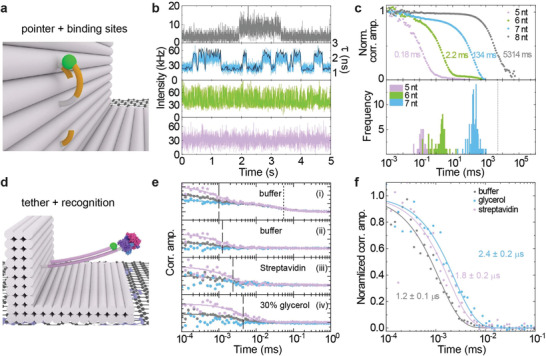
Dynamic DNA origami nanostructures studied with GET. a) Sketch of the L‐shaped DNA origami structure with a flexible pointer with fluorescent dye (Cy3B), and upper (26.5 nm) and lower (16.1 nm) binding strands yielding GET efficiencies of 15.7% (τ_up_ = 2.6 ns) and 59.3% (τ_low_ = 1.3 ns). b) Representative transients for 8 (gray), 7 (blue), 6 (green), and 5 (lilac) nt binding. For 7 nt binding, the fluorescence lifetime (black) is also shown with 20 ms binning. All transients were acquired at 3 µW excitation power, except for eight nt binding (1 µW). c) Normalized correlation functions averaged over several transients and corresponding frequency distribution resulting from analyzing each transient individually. The gray dashed vertical line represents the correlation time for 8 nt extracted from concatenated transients. d) Sketch of the biosensing system with a 44 nt long tether, Cy3B, and a target recognizing unit (biotin) and target (streptavidin). e) Averaged correlation functions for photons with a long microtime (>2.5 ns) for measurements of the tether fluctuations in buffer (gray), in buffer with 30% glycerol (blue) and in buffer, incubated with streptavidin (lilac) after subtracting the fit of the correlation functions calculated from photons with a short microtime (<2.5 ns). See Supporting Information for a detailed description of the gating procedure.

Next, we studied whether dynamic distance changes to graphene can be visualized by GET. We used the L‐shaped DNA origami structure equipped with a Cy3B labeled 19 nucleotides (nt) single‐stranded DNA pointer which can transiently bind to two protruding strands, ≈6 nm below and above the pointer position (see sketch in **Figure** **2**a). L‐shaped DNA origami structures with correct orientation exhibit strong fluctuations in the fluorescence intensity due to dynamic switching of the binding position between lower (strong quenching) and upper (weak quenching) strand. Figure 2b shows exemplary transients with decreasing strength of the binding interactions from 8 (gray) to 5 (lilac) complementary nucleotides. With 8 nt binding, switching between the two bound conformations occurs on the time scale of seconds as visualized by the two intensity levels (Figure 2b, gray). With 7 nt binding (blue), frequent transitions occur in the hundred millisecond range. For this example, a fluorescence lifetime transient is also depicted (black) verifying that intensity fluctuations are directly correlated with the fluorescence lifetime. For 6 and 5 nt (green and lilac), the transitions are too fast to be visually resolved in the transients but they are revealed by autocorrelation analysis. The correlation functions averaged over several transients are displayed in Figure 2c, together with the correlation time distribution for the different number of nucleotides per binding strand (see Supporting Information for details of autocorrelation). As the correlation time for 8 nt binding is too long to extract it from a single transient with sufficient statistical accuracy, only a single value (vertical dashed line) is given which results from concatenating all acquired transients.

We have further advanced this approach for dynamic sensing of confined diffusion changes of a flexible 44 nt double stranded tether (see sketch in Figure 2d). As the tether is able to perform a confined diffusion around the point of attachment, the distance between the Cy3B dye at the end of the tether and the graphene surface is permanently changing which causes fast intensity fluctuations. As this intensity fluctuation is directly correlated to the fluorescence lifetime, it can be distinguished from other sources of photophysical intensity fluctuations such as triplet states by correlating different subsets of photons depending on their arrival time with respect to the laser pulse (see Supporting Information for details). The resulting filtered component for tether movements on graphene (Figure 2e, averaged over several molecules) is sensitive to changes of the diffusion properties of the tether and can be used to detect binding events (streptavidin to biotin at the end of the tether (Figure 2e, lilac) as well as viscosity changes (buffer with 30% glycerol in Figure 2e, blue). Changes of the correlation time from 1.2 to 1.8 and 2.6 µs, respectively, are revealed in analogy to confocal fluorescence correlation spectroscopy (see Supporting Information for comparison and further details on analysis).

### 2.3 Expanding FRET

**Figure 3:**
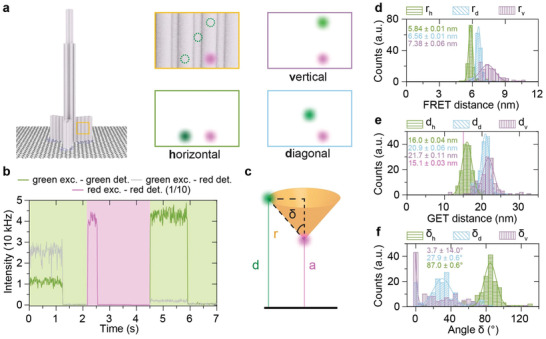
Static FRET/GET measurements. a) Illustration of the pillar‐shaped DNA origami structure with three different acceptor–donor orientations: horizontal h, diagonal d, and vertical v. ATTO542 and ATTO647N were used as a donor and acceptor dye, respectively. b) Exemplary transient of the acceptor bleaching approach for the horizontal sample on glass. The background colors indicate the excitation laser (green: 532 nm, magenta​: 640 nm). First donor (green) and FRET (gray) signal can be observed. Around 1.2 s, the green laser is switched off and the red laser is switched on (2.1 s, shown acceptor count rate is 1/10 of detected count rate) until the acceptor (magenta) bleaches (2.6 s). Green laser excitation is then used until the donor bleaches. c) Illustration of parameters accessible by FRET/GET. d) Distributions of the distance between donor and acceptor *r* calculated from the FRET data measured on glass. e) The distribution of distances of donor *d* to graphene, including the average acceptor–graphene distance (magenta line). f) The distributions of the angle δ calculated from the results presented in (d) and (e). All errors are standard errors from Gaussian distributions, besides the errors of the delta which are calculated from the error propagation.

FRET is a workhorse of single‐molecule biophysics.^[^ ^22^, ^23^ ^]^ FRET experiments require labeling with a donor and acceptor fluorophore with a distance up to 10 nm. In GET, an unbleachable, broad‐band acceptor “molecule” is provided complimentary without requiring an additional labeling. GET provides the distance of the dye (acceptor and donor) to graphene. From FRET, the distance between acceptor and donor is determined. Next, we explored whether combining FRET and GET could yield additional information such as the orientation of the FRET‐pair with respect to the surface. To this end, we prepared three DNA origami constructs that exhibit similar FRET efficiency but different orientation of the FRET‐pair with respect to the surface (**Figure** **3**a).

The arrangements are termed vertical (v), horizontal (h), and diagonal (d). We determined accurate FRET efficiency *E* using the acceptor bleaching approach in fluorescence transients.^[^ ^24^ ^]^ Therefore, we first excited the donor and recorded fluorescence intensities of the green and red channel (see transients in Figure 3b, green—donor; gray—FRET). Around 1.2 s the green excitation was switched off, and at 2.1 s, we switched to red excitation and photobleached the acceptor. Finally, we probed the green emission with green excitation to obtain the intensity of the donor in the absence of the acceptor. With this data, we calculated the FRET distance ***r***, the distances to graphene *d* and *a*, and the angle δ (see Figure 3c). The FRET efficiency was obtained from the donor lifetime in presence and absence of the acceptor (see Figure S4, Supporting Information). Under the assumption of isotopically rotating dipoles (κ^2^ = 2/3), the FRET distance was calculated from measurements on glass (Figure 3d) as graphene might slightly change the FRET rate constant.^[^ ^25^, ^26^, ^27^, ^28^ ^]^

Next, the distances to graphene (GET distance) were calculated from the acceptor lifetime and the donor lifetime after acceptor bleaching. The position of the acceptor was fixed (magenta line in Figure 3e at 15.1 nm) whereas the donor position was varied (Figure 3e; Supporting Information for details). Finally, based on the results of FRET and GET distances, the angle δ (δ_h_, δ_d_, δ_v_) was calculated (Figure 2f) directly visualizing the additional information obtained by GET–FRET. The angles of the vertical (4° ± 14°), diagonal (28° ± 0.6°) and horizontal samples (87° ± 0.6°) are close to the designed values of 0°, 37°, and 90°. The slight differences to the designed angles are related to the limited accuracy of our design model and might also be caused by linker lengths and preferred orientations and interactions of the dyes with the DNA.

**Figure 4:**
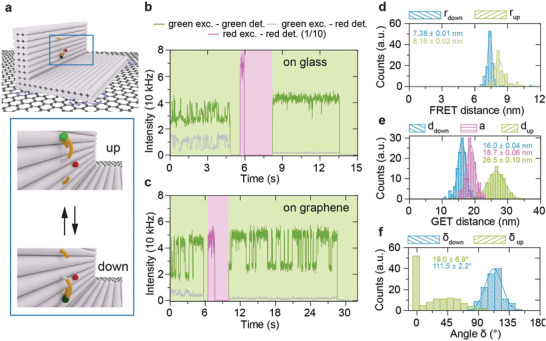
Dynamic FRET/GET measurements. a) Illustration of the L‐shaped DNA origami structure featuring a pointer which can transiently bind to an upper and a lower binding site. ATTO542 and ATTO647N were used as a donor and acceptor dye, respectively. b,c) Transients of the FRET assay on glass (b) and graphene (c). The glass transient only shows FRET‐based fluctuation in presence of the acceptor while the graphene transient show either GET‐based fluctuation after acceptor bleaching or combined FRET‐ and GET‐based fluctuation in the presence of the acceptor. d) The FRET distances (*r*_down_, *r*_up_) calculated from the FRET measurements on glass and e) the GET distances (*d*_down_, *a*, *d*_up_). f) From the combined results of d,e) the angles (δ_down_, δ_up_) were determined. All errors are standard errors from Gaussian distributions, besides the errors of the delta, which are calculated from the error propagation.

Single‐molecule FRET is especially valuable for visualizing dynamic processes. We used the same L‐shaped DNA origami structure as described in the previous section with a pointer that transiently hybridizes to two short oligonucleotides and added an acceptor dye closer to the lower binding position, in two binding modes, either the “up” mode where low GET and FRET is observed or the “down” mode with high GET and FRET (see sketch in **Figure** **4**a). The sample on glass only shows a modulation in presence of the acceptor (until 5 s, Figure 4b), after acceptor bleaching the modulation disappears. On the graphene sample, modulation in presence and absence of the acceptor (Figure 4c) is observed. During the first excitation with green (until 6 s) the modulation is caused by the combined influence of FRET and GET, after the acceptor bleached the modulation is caused only by GET. From the FRET data on glass, the FRET distance was calculated (Figure 4d). Combining FRET data and GET data (Figure 4e) enabled determining the orientation in space (Figure 4f). The directions of FRET for binding to the lower and upper position exhibit an angle of 112° and 19°, respectively.

### 2.4 Graphene Biosensing

**Figure 5:**
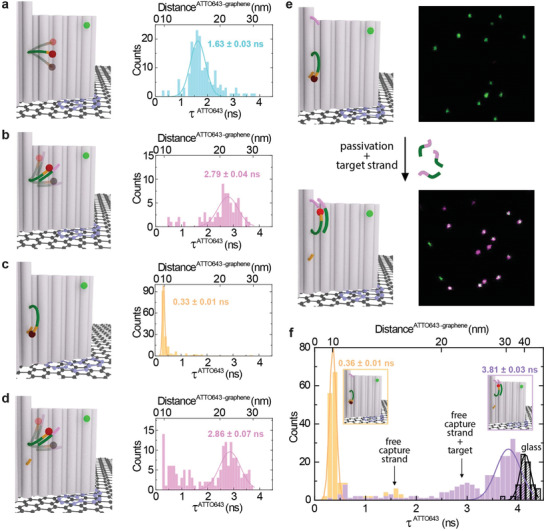
Graphene biosensing with a nucleic acid bioassay. a–d) Sketches and histograms of the fluorescence lifetime distributions fitted with a Gaussian function (the mean value and standard error obtained from the fit) of an ATTO643 dye attached to the capture strand which is: a) free to move (cyan), b) bound to a target strand (magenta), c) hybridized with a closing strand (orange), and d) liberated by a target strand (magenta). e) Demonstration of the full assay: sketches and fluorescence intensity maps (10 × 10 µm), on the top—before a target detection (a capture strand hybridized with a closing strand), on the bottom—a capture strand liberated by a target strand and additionally caught and stabilized by a biasing strand. f) Histogram of the fluorescence lifetime distributions fitted with Gaussian functions (the mean value and standard error obtained from the fit) of an ATTO643 dye in the full assay before (orange) and after (lilac) target detection; in black, results for the assay measured on glass.

Fluorescence quenchers are often employed in biosensing assays in which a binding event yields a change of the dye–quencher interaction so that the fluorescence change is indicative of a binding event. For using graphene as quencher in nucleic acid biosensing assays, we started from the pillar‐shaped DNA origami nanostructure (see Figure 3a) and equipped it with a dye‐labeled protruding capturing sequence at a height of 16.3 nm (ATTO643 at the 3′ end), i.e., close to *d*_0_‐value at which the fluorescence intensity is most sensitive to height changes. An ATTO542 dye in the DNA origami structure at a height of 23.4 nm from graphene served as internal reference (**Figure** **5**, sketches, Supporting Information for details on data analysis). The dye on the capture strand exhibits an intermediate fluorescence intensity with multiexponential fluorescence decays (Figure S6a, Supporting Information) and a maximum of the fluorescence lifetime distribution at 1.63 ± 0.03 ns (Figure 5a) when approximated by a single exponential decay.

Multiexponential fluorescence decays from single molecules can occur when the dye labeled strand is sampling different dye–graphene distances during the measurement. Upon hybridization with the target strand, a change of the fluorescence properties and a main population with a fluorescence lifetime around 2.79 ± 0.04 ns is observed (Figure 5b; Figure S6b, Supporting Information). We found that this increase in average fluorescence lifetime stems from an interaction of the target molecule with the DNA origami structure (Figure S8, Supporting Information). To increase the contrast, we inserted another protruding strand with 12 nt complementary to the capture strand (closing strand at 9.2 nm), which is closer to the bottom of the DNA origami structure (see Figure 5c). In the absence of target, the capturing strand binds to the closing strand yielding strongly quenched dyes with a fluorescence lifetime of 0.33 ± 0.01 ns, i.e., 92% ± 1.1% quenching efficiency (Figure 5c). Opening of this closed conformation by the target occurs via a 12 nt toehold on the capture strand and a similar fluorescence lifetime distribution was obtained as before with only a small fraction of capturing strands remaining bound to the closing strand (compare Figure 5b with Figure 5d). To further increase the contrast by maximizing the signal of the open form, we added another capture region that is able to bind the target DNA away from the graphene surface (lilac strand in Figure 5e protruding at a height of 30.6 nm above graphene). This additional capture sequence binds to a part of the target that is not binding to the capture strand and it is designed so short (9 nt) that it imposes a strong pointing bias without creating a thermodynamic trap (therefore the strand is denoted “biasing strand” in the following). The short biasing strand ensures that initially the target hybridizes to the capture strand, replaces it from the closing strand and forms a new loop with the biasing strand thereby restoring the fluorescence of the dye ATTO643. Initially, the fluorescence of this construct is quenched in analogy to the sample without biasing strand (Figure 5c, compare fluorescence decays in Figure S6c,e, Supporting Information). Upon target binding, we observed almost full unquenching of the fluorescence (compare lilac population in Figure 5f with glass reference in black) with minor fractions of strong quenching (τ < 0.7 ns, hybridization of capture strand with closing strand) and mild unquenching (2.3 ns < τ < 3.3 ns, target binding but not binding to biasing strand). Overall, we could stepwise increase our signal contrast upon target binding indicating the potential of designing assays with DNA origami nanopositioners on graphene.

### 2.5 GET Tracking

**Figure 6:**
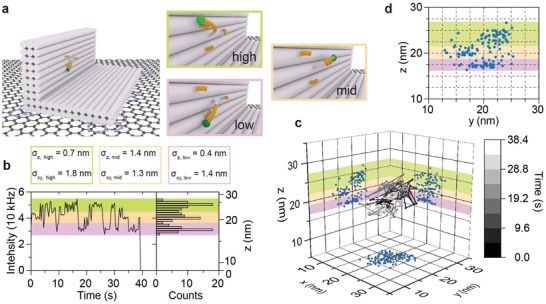
GET tracking. a) Sketch of the L‐shaped DNA origami structure with three protruding strands “low” (light violet), “mid” (orange), and “high” (green), to which the Cy3B dye‐labeled pointer can transiently hybridize. b) Intensity transient for a single pointer imaged by TIRF microscopy. An intensity histogram is shown to the right of the transient. The three intensity levels are representing the transient hybridization of the pointer to the protruding strands. c) 3D trajectory of the pointer extracted from the fluorescence intensities (*z*‐resolution) and from fitting of the point‐spread‐functions (*xy*‐resolution). Localization precision was between 1.3 and 1.8 nm in *xy* and between 0.4 and 1.4 nm in the *z*‐direction. d) An inset of the *y*/*z* projection which clearly shows the well‐resolved binding sites.

Camera‐based localization methods are used to track single molecules in two dimensions and scanning a confocal spot with feedback enables recording 3D trajectories of single molecules.^[^ ^29^, ^30^ ^]^ Achieving isotropic nanoscale resolution in three dimensions, however, remains a challenge. Here, we show 3D tracking of a dye‐labeled DNA pointer that can transiently hybridize to three single‐stranded protrusions on the L‐shaped DNA origami structure. Two of the three protruding strands are arranged vertically at a distance of two helices (≈6 nm) (see sketches in **Figure** **6**a). The height of the third protruding strand is in the middle between the other two strands and displaced to the side by ≈5.4 nm. With respect to graphene, the three binding sites are at heights of 24 nm (high), 21 nm (mid), and 18 nm (low).

From TIRF imaging of the Cy3B‐labeled pointer, we extracted intensity transients as the one depicted in Figure 6b. Three intensity levels representing binding to the different protruding strands are reflected in the intensity histogram shown next to the transient. Combining *xy*‐information from fitting the point‐spread function with the intensity *z*‐information yields tracking trajectories such as those shown in Figure 6c and Figure S9 (Supporting Information). The *y*/*z* projection (Figure 6d) shows that all three binding positions are clearly resolved. Fitting the three populations independently yields a localization precision of 0.4 to 1.4 nm in *z*‐direction and 1.3 to 1.8 nm in *xy*‐direction. Such molecular precision tracking at small length scales should be able to complement single‐molecule FRET, as 3D information is obtained over an extended distance range in contrast to distances only (experimental details in the Supporting Information).

### 2.6 GET‐DNA PAINT Super‐Resolution

Surface quenching was first used for super‐resolution microscopy with metal‐induced energy transfer to gold surfaces.^[^ ^31^, ^32^ ^]^ The steeper distance dependence of graphene quenching compared to gold quenching enables the determination of the *z*‐distance of molecules from the surface with improved precision and has been used to determine the distance law of graphene quenching and the thickness of lipid membranes.^[^ ^9^, ^17^ ^]^ In addition, graphene creates less background and fluorescence detection can be comfortably carried out through the graphene‐on‐glass coverslip. Here, we explored whether graphene quenching is compatible with single‐molecule localization super‐resolution microscopy by DNA PAINT,^[^ ^33^, ^34^, ^35^ ^]^ in which a DNA origami structure is super‐resolved by successively visualizing the binding events of single molecules to the DNA‐labeled structure of interest.

**Figure 7:**
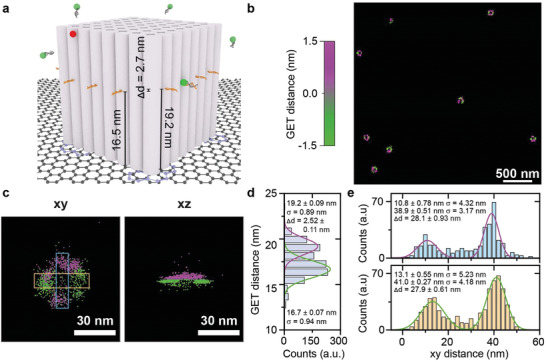
GET‐super‐resolution on a cubic DNA origami structure with DNA PAINT. a) Sketch of the DNA origami cube with DNA PAINT docking sites. Opposing sides have equal height with a difference of 2.7 nm between the adjacent sides. ATTO647N was used to monitor the DNA origami structure density. b) Super‐resolution image of DNA origami cube with a heatmap. c) *x*/*y* and *x*/z projections of the DNA origami cube with a different height indicated by the same heatmap as in (b). d) The cross‐section along the *z*‐direction shows a resolution of 2.5 nm with a localization precision between 0.89 and 0.94 nm. e) Cross‐sections along the *x*‐axis (blue histogram) and the *y*‐axis (orange histogram) in the *xy* plane with a localization precision between 3.2 nm and 5.2 nm.

We equipped a cube‐shaped DNA origami structure (obtained from GATTAquant) with DNA PAINT binding sites. On two opposing sides of the DNA cube with a side length of 24 nm, we placed 8 nt binding sites at a height of 19.2 nm above graphene. On the other two opposing sides, we placed binding sites at a height of 16.5 nm (**Figure** **7**a). For DNA PAINT imaging, we used an 8 nt long ATTO542 labeled imager strand. We generated 2D images via fitting of the point‐spread function and determined the *z*‐position from the fluorescence intensity excluding the first and the last frame of each binding event (see the Supporting Information for details on experimental procedures and data analysis). An overview image of the DNA origami cubes is shown in Figure 7b, in which the height information is color‐coded. The exemplary magnified views of the *x*/*y* and *x*/*z* projections (Figure 7c–e; Figure S10, Supporting Information) show that the structure is resolved in *xy* (σ ≈ 5 nm) and in *z* with a demonstrated resolution better than 3 nm. To the best of our knowledge, this is the finest structural detail in the axial direction that has been resolved by optical microscopy. Another advantage of measuring DNA PAINT on graphene is that unspecific surface binding of imagers goes along with complete quenching instead of creating unspecific localizations.

## 3 Conclusion

We have introduced graphene‐on‐glass coverslips as a novel platform for single‐molecule biophysics, biosensing, and super‐resolution microscopy. Graphene represents a broadband, unbleachable energy transfer acceptor that is transparent for imaging and even reduces background by quenching unspecifically bound molecules. Using DNA origami structure nanopositioning, we carried out a series of assays with several unique and innovative abilities including the detection of the angle of a FRET pair as well as its distance with respect to a surface. GET tracking as a dynamic tool for super‐resolution enables isotropic precision down to the molecular range. The combination of DNA PAINT with visualized structural details of 2.5 nm in *z*‐direction provided by graphene quenching enables unique resolution. In combination with novel imaging modalities such as p‐MINFLUX^[^ ^36^, ^37^ ^]^ and the potential to synergistically exploit graphene's electrical properties, graphene energy transfer opens new windows for single‐molecule biophysics, biosensing, and super‐resolution with exquisite resolution close to the coverslip.

## Conflict of Interest

The invention entitled “Method of immobilization nucleic acid structure on surface of graphene, graphene modified by immobilization of nucleic acid structure and its use” has been submitted to European Patent Office (Authors: Dr. Izabela Kaminska and Prof. Dr. Philip Tinnefeld), number EP19461514.2. Prof. Dr. Philip Tinnefeld is consulting and mentoring the GATTAquant team.

## Supporting information

Supporting Information

Supplemental Video 1

Supplemental Video 2

Supplemental Video 3

## Data Availability

Research data are not shared.
