# Supplementary material for: Graphene Energy Transfer for Single‐Molecule Biophysics, Biosensing, and Super‐Resolution Microscopy
Source: Adv Mater. 2021 May 3;33(24):2101099. doi: 10.1002/adma.202101099 (PMC11468539; doi:10.1002/adma.202101099)
Supplement: Supplementary file 1 — Supporting Information [file ADMA-33-2101099-s001.pdf]

© 2021 Wiley-VCH GmbH

ADVANCED MATERIALS

Supporting Information

for *Adv. Mater.,* DOI: 10.1002/adma.202101099

Graphene Energy Transfer for Single-Molecule Biophysics, Biosensing, and Super-Resolution Microscopy

Izabela Kamińska, Johann Bohlen, Renukka Yaadav, Patrick Schüler, Mario Raab, Tim Schröder, Jonas Zähringer, Karolina Zielonka, Stefan Krause, and Philip Tinnefeld**

Supporting Information

Graphene Energy Transfer for Single-Molecule Biophysics, Biosensing & Superresolution Microscopy

Izabela Kamińska, Johann Bohlen, Renukka Yaadav, Patrick Schüler, Mario Raab, Tim Schröder, Jonas Zähringer, Karolina Zielonka, Stefan Krause, Philip Tinnefeld**

Table of Contents

1. Materials and Methods 2
1.1. Buffers and recipes 2
1.2. Dye molecules 2
1.3. Preparation of DNA origami structures 3
1.4. Preparation of graphene-on-glass coverslips 4
2. Imaging and Analysis 5
2.1. Fluorescence confocal microscope I 5
2.2. Fluorescence confocal microscope II 5
2.3. TIRF microscope I 6
2.4. TIRF microscope II 7
3. Experiments 7
3.1. Distance determination from fluorescence lifetimes. 7
3.2. Dynamics with GET. 9
3.3. Expanding FRET. 14
3.4. Graphene biosensing 21
3.5. GET tracking. 24
3.6. GET-DNA PAINT superresolution. 26
4. DNA origami structure design 29
5. DNA sequences 30
6. References 49

1. Materials and Methods

1.1. Buffers and recipes

If no other company is mentioned, chemicals were purchased from Sigma Aldrich.

Table S1. The list of buffers with recipes.

Name	Recipe
FOB20	20 mM $MgCl_2 \cdot 6H_2O$<br>20 mM Tris base<br>20 mM acetic acid<br>1 mM EDTA-$Na_2 \cdot 2\ H_2O$
FOB12.5	12.5 mM $MgCl_2 \cdot 6H_2O$<br>20 mM Tris base<br>20 mM acetic acid<br>1 mM EDTA-$Na_2 \cdot 2\ H_2O$
PCA/Trolox12	2 mM Trolox (6-hydroxy-2,5,7,8-tetramethylchroman-2-carboxylic acid)<br>25 mM PCA (protocatechuic acid)<br>12 mM $MgCl_2 \cdot 6H_2O$<br>40 mM Tris base<br>20 mM acetic acid<br>1 mM EDTA-$Na_2 \cdot 2H_2O$
PCA/Trolox2	2 mM Trolox (6-hydroxy-2,5,7,8-tetramethylchroman-2-carboxylic acid)<br>25 mM PCA (protocatechuic acid)<br>2 M NaCl<br>40 mM Tris base<br>20 mM acetic acid<br>1 mM EDTA-$Na_2 \cdot 2H_2O$
50× PCD	2.8 mM PCD (protocatechuate 3,4-dioxygenase from pseudomonas sp.)<br>50% glycerol<br>50 mM KCl<br>100 mM Tris HCl<br>1 mM EDTA-$Na_2 \cdot 2H_2O$
Glox12	2mM aged Trolox<br>1% glycerin<br>12.5 mM $MgCl_2 \cdot 6H_2O$<br>1× TAE<br>0.8 µM Glucose oxidase<br>0.04% Catalase

1.2. Dye molecules

Table S2. Dyes used in all experiments and the properties which determined their selection. All dye molecules are suitable for single-molecule applications and high-resolution microscopy. More information can, e.g. be found on the manufacturer's web representation and in refs.[1–3]

Dye molecule	Sections/experiments	Properties
ATTO647N	- Distance determination from fluorescence lifetimes.	- fluorescence label in the red spectral region,<br>- exceptionally high photostability,

	- Expanding FRET.<br>- Colocalization dye in GET superresolution.	- absorption and fluorescence independent of pH,<br>- cationic dye, which may stick to negatively charged DNA.
ATTO643	- Distance determination from fluorescence lifetimes/orientation of L-shaped DNA origami structure.<br>- Graphene biosensing.	- similar properties as ATTO647N,<br>- significantly reduced tendency for unspecific binding,<br>- anionic dye, does not stick to negatively charged DNA,<br>- recommended for dynamic experiments.
Cy3B	- Dynamics with GET.<br>- GET tracking.	- fluorescence label in the orange spectral region (560 nm)<br>- bright dye in combination with PCA/PCD<br>- negligible blinking with oxygen scavenging and ROXS<br>- cationic dye, which may stick to negatively charged DNA.
ATTO542	- Expanding FRET.<br>- GET superresolution.<br><br>Colocalization dye in:<br>- Distance determination from fluorescence lifetimes;<br>- Graphene biosensing.	- fluorescence label in the green spectral region,<br>- high photostability and high fluorescence quantum yield,<br>- very suitable for oligonucleotide labeling, used in both static and dynamic experiments (non-sticky).

1.3. Preparation of DNA origami structures

The cube-shaped DNA origami structure was purchased from GATTAquant®. The other DNA origami nanostructures used were designed in caDNAno and utilized the p8064 scaffold derived from M13mp18 bacteriophages. DNA origami structures were folded with a 10-fold excess of unmodified and internally labeled oligonucleotides and a 100-fold excess of biotinylated or pyrene-modified oligonucleotides in comparison to the scaffold in 1× FOB20 buffer. The details of the folding program can be found here[4]. After folding, 1× Blue Juice gel loading buffer was added to the DNA origami solution which was then purified via agarose-gel electrophoresis with 1.5% agarose gel in 50 mL of FOB12.5 buffer (details in Table S2). The specific band for the nanostructure was extracted from the gel. Before putting the purified DNA origami solution onto glass or graphene, the concentration was adjusted with FOB12.5 buffer to 25 pM (GET tracking), 800 pM (GET-DNA PAINT superresolution) or 50 pM (all the other experiments).

Table S3. Agarose-gel electrophoresis protocols for each experiment.

Experiment	peqGREEN (VWR) µL/100 µL of buffer	Voltage [V]	Time [h]
Distance determination from fluorescence lifetimes	2	80	1
Dynamics with GET	2	80	1
Expanding FRET	none	60	2
Graphene biosensing	2	60	1.5
GET tracking	none	60	2

Positions and distances of dyes in DNA origami structures were estimated assuming a distance of 0.34 nm between the nucleotides along the DNA double helix and 2.7 nm between the centers of adjacent helices, in a square lattice.[5,6] Additionally, 1 nm was added to include the presence of pyrene molecules incorporated via external labeling.[7] Any deviations from the estimated values may stem from bending or tilting of DNA origami structures.[8] It also has to be taken into account that differences between the designed and measured distances may depend on the specific structure, dye-DNA interactions and salt concentration.

1.4. Preparation of graphene-on-glass coverslips

Monolayer graphene on a 60 mm × 40 mm copper substrate with poly(methyl methacrylate)(PMMA) on top was purchased from Graphenea®. Subsequently, a wet-transfer approach was used to transfer the graphene to glass coverslips.[9] All coverslips were treated with UV-Ozone cleaning at 100 ˚C for 30 minutes on each side to remove any contaminants from the surface. Smaller pieces of roughly 0.25 cm² were carefully cut from the PMMA/Gr/Cu foil. The copper was wet-etched by letting a piece float with the copper film exposed to 0.2 M ammonium persulfate for ~4 hours. A coverslip was dipped vertically while slowly moving towards the PMMA/Gr and scooped it gently out of the solution and transferred to milliQ water in order to wash out the residues of ammonium persulfate.[9] The step of washing PMMA/Gr was repeated twice with fresh milliQ water. Next, the PMMA/Gr was scooped with a glass coverslip and carefully dried using nitrogen stream. Another layer of liquid PMMA (M_w = 120,000 g/mol) dissolved in chlorobenzene (50 mg/mL) was drop-casted on top of the first PMMA/Gr layer. This allowed the dried PMMA to re-dissolve thus relaxing the underlying graphene monolayer and forming an improved contact with the substrate.[10] After 30 minutes, the PMMA/Gr on glass was first dipped in acetone for 5-10 min., then in toluene for 5-10 min., and again in fresh acetone for 5-10 min. After each washing step, in acetone or toluene, the

samples were dried with a nitrogen stream. Finally, the sample was placed on active coal, heated on a heating plate to 230˚C, for 30 minutes, and then left to cool down.

2. Imaging and Analysis

2.1. Fluorescence confocal microscope I

Single-molecule fluorescence measurements (Distance determination from fluorescence lifetimes and Graphene biosensing) were performed on a custom-built confocal microscope I, based on an inverted microscope (IX-83, Olympus Corporation, Japan) and a 78 MHz-pulsed supercontinuum white light laser (SuperK Extreme, NKT Photonics A/S, Denmark) with selected wavelengths of 532 nm and 639 nm. The wavelengths are selected via an acousto-optic tunable filter (AOTF, SuperK Dual AOTF, NKT Photonics A/S, Denmark). This is controlled by a digital controller (AODS 20160 8R, Crystal Technology, USA) via a computer software (AODS 20160 Control Panel, Crystal Technology, Inc., USA). A second AOTF (AA.AOTF.ns: TN, AA Opto-Electronic, France) was used to alternate 532 nm and 639 nm wavelengths, as well as to further spectrally clean the laser beam. It is controlled via self-written LabVIEW software (National Instruments, USA). A neutral density filter was used to regulate the laser intensity, followed by a linear polarizer and a λ/4 plate to achieve circularly polarized excitation. A dichroic beam splitter (ZT532/640rpc, Chroma Technology, USA) and an oil immersion objective (UPlanSApo 100×, NA = 1.4, WD = 0.12 mm, Olympus Corporation, Japan) were used to focus the excitation laser onto the sample. Nanopositioning was performed using a Piezo-Stage (P-517.3CL, E-501.00, Physik Instrumente GmbH&Co. KG, Germany). The excitation powers at both 532 and 639 nm were set to 1 μW. Emitted light was collected using the same objective and filtered from the excitation light by the dichroic beam splitter. The light was later focused on a 50 μm pinhole (Linos AG, Germany) and detected using Single-Photon Avalanche Diodes (SPCM, AQR 14, PerkinElmer, Inc., USA) registered by a TCSPC system (HydraHarp 400, PicoQuant GmbH, Germany) after additional spectral filtering (RazorEdge 647, Semrock Inc., USA for the red channel and BrightLine HC 582/75, Semrock Inc., USA for the green channel). A custom-made LabVIEW software (National Instruments, USA) was used to process the acquired raw data.

2.2. Fluorescence confocal microscope II

Single-molecule fluorescence measurements (Dynamics with GET and Expanding FRET) were performed on another home-built confocal setup II based on an Olympus IX71 microscope. The green laser (LDH-P-FA-530B, Picoquant) is controlled by a PDL 828 “Sepia II” (Picoquant).

The green fiber (polarisation maintaining fiber with FC/APC output connector) coupled laser light is decoupled via a F220APC-532 collimator (Thorlabs) and cleaned up with a 532/2 (Z532/10 X, Chroma) filter before passing a dichroic mirror (640 LPXR, Chroma) for optional combination with the already cleaned up (Z640/10 X, Chroma) red laser (LDH-D-C-640, Picoquant). A linear polarizer (WP12L-Vis, Thorlabs) and a quarter-wave plate (AQWP05M-600, Thorlabs) are combined to obtain circularly polarized light. After passing a second dichroic mirror (zt532/640rpc, Chroma) the beam is focused via an oil immersion objective (UPLSAPO 100 XO, NA 1.40, Olympus) onto the samples. The sample is scanned with a piezo-stage (P-527.3CD, Physik Instrumente) which is controlled by an E-727 controller (Physik Instrumente). The emitted light is focused on a 50 µm pinhole (Thorlabs) and collimated with a lens (AC050-150-A-ML, Thorlabs).

For the correlation sample, the beam is cleaned with a filter set (582/75 BrightLine HC, Semrock and LP03-532RU-25, Semrock) before it is split by a 50:50 non-polarizing beam splitter cube (BS013, Thorlabs).

For FRET experiments, the beam is split with a dichroic mirror (640 LPXR, Chroma) and cleaned with a filter set (red: FESH0750, Thorlabs and LP02-647RU-25, Semrock green: 582/75 BrightLine HC, Semrock and LP03-532RU-25, Semrock). Afterwards, the beam is focused via lenses (red: AC080-020-B-ML; green: AC080-020-A-ML, Thorlabs) on two APDs (SPCM-AQRH-TR-14, Excelitas). The APDs' signal is processed with a HydraHarp 400 (PicoQuant) and controlled with the software SymPhoTime 64 (PicoQuant). Further data analysis is performed with self-written Matlab and LabVIEW routines.

2.3. TIRF microscope I

In the home-built widefield/ TIRF (total internal reflection fluorescence) microscope, a 644 nm diode laser (150 mW, iBeam smart, Toptica Photonics) and a 560 nm fiber laser (1 W, MPB Communications) are exciting the samples. After both lasers are cleaned up with filters (644 nm: Brightline HC 650/13, Semrock; 560 nm: Brightline HC 561/4, Semrock) the beams are combined with a dichroic mirror (T612lpxr, Chroma). To expand the beam profile, the laser passes through lenses (Bi-convex f50, Thorlabs; AC f120, Linos). In the microscope body (IX 71, Olympus) the beam passes a dichroic mirror (z476-488/568/647, Chroma) and is focused by an objective (100×, NA =1.4, UPlanSApo, Olympus). To avoid drift, the objective is mounted on a nose piece (IX-2NPS, Olympus). The emitted light is collected by the same objective and passes a 1.6× optical lens and an emission filter (644 nm: ET 700/75, Chroma or 560 nm: Brightline HC 561/4, Chroma). Images and movies are recorded by an EMCCD

(electron multiplying charge-coupled device) camera (iXon+ 3384, Andor). The lasers are operated with Topas iBeam smart software (Toptica Photonics, 644 nm), GUI-VFL software (MPB Communications, 560 nm) and the camera with ImageJ plugin Micro-Manager 1.4.[11]

2.4. TIRF microscope II

The second widefield/TIRF microscope is powered by a 644 nm diode laser (150 mW, ibeam smart, Toptica Photonics) and a 532 nm fiber laser (1W, MPB Communication). After both lasers are cleaned up (644 nm: Brightline HC 650/13, Semrock; 532 nm z532/647x, Chroma) and aligned, the beam is directed over a dichroic mirror (Dual Line zt532/640 rpc, AHF Analysentechnik) to the back focal plane of the objective (UPLXAPO 100×, NA = 1.45, WD = 0.13, Olympus). The microscope body (IX71, Olympus) is equipped with a nose piece (IX2-NPS, Olympus) and put on an actively stabilized optical table (TS-300, JRS Scientific Instruments) to stabilize the sample. The emission is cleaned up (644 nm: ET 700/75, Chroma; 532 nm: BrightLine 582/75, AHF Analysentechnik) and detected on an EMCCD camera (iXon X3 DU-897, Andor). The lasers are operated with Topas iBeam smart software (Toptica Photonics, 644 nm), GUI-VFL software (MPB Communications, 532 nm) and the camera with ImageJ plugin Micro-Manager 1.4.[11]

3. Experiments

3.1. Distance determination from fluorescence lifetimes.

All measurements for this part were carried out on the confocal setup I (SI chapter 2.1) with a pulsed interleaved laser excitation of 532 nm and 639 nm. Three samples of a pillar-shaped DNA origami structure were labeled with a green (ATTO542) and a red (ATTO647N) dye molecule at the heights of 15.9 and 52.5 nm (**1**), 11.6 and 23.4 nm (**2**), 15.9 and 15.9 nm (**3**), respectively. Each DNA origami nanostructure was immobilized either on neutravidin–biotinylated BSA glass coverslips using biotin modification at the base of the structure, or they were immobilized on graphene using pyrene modification. Single molecules were identified in the fluorescence images and their fluorescence lifetime was determined. Figure S1a depicts the scatter plot and corresponding histograms of the obtained fluorescence lifetime values for the measurements performed on glass (∇) or graphene (□, +, ○). All three populations of the nanostructures measured on graphene are clearly distinguishable. The distributions of the fluorescence lifetime are slightly broadened on graphene compared to glass, directly indicating minimal heterogeneity in the DNA origami structure immobilization. We can attribute this heterogeneity to several factors, including, e.g., tilting or structural heterogeneity of the DNA

origami structure, less photons recorded in the presence of graphene, as well as impurities or defects of graphene substrate.[12] Nevertheless, the differences are not substantial, and most importantly the three populations are well-separated.

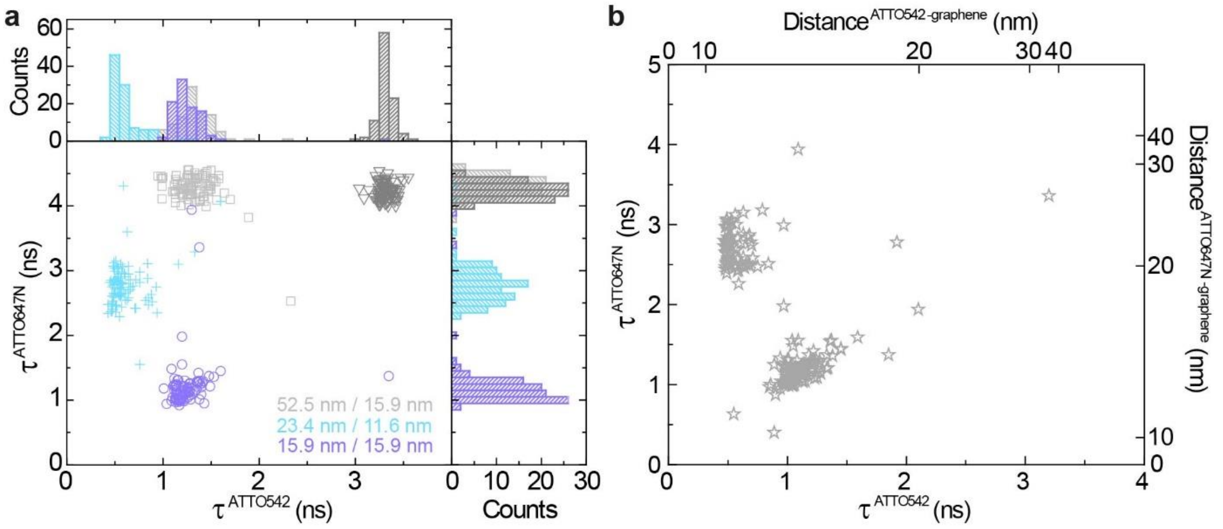

Figure S1. Distance determination from fluorescence lifetimes. (a) Scatter plot and corresponding histograms of fluorescence lifetime of colocalized dye molecules at a pillar-shaped DNA origami structure (both dye molecules within one DNA origami structure): ATTO542 and ATTO647N, at various heights; each DNA origami sample measured separately on glass (∇) or graphene (□, +, ○), with ATTO542/ATTO647N at 15.9/52.5 nm (□ grey), 11.6/23.4 nm (+ cyan) or 15.9/15.9 nm (○ violet) distance from graphene. (b) Scatter plot of fluorescence lifetime of colocalized dye molecules at a pillar-shaped DNA origami structure: two mixed DNA origami structures with ATTO542 and ATTO647N dyes molecules at the height of 11.6/23.4 nm and 15.9/15.9 nm above graphene, imaged together on one graphene substrate.

Next, samples (**2**) and (**3**) were mixed, immobilized on graphene together and imaged. Again, single molecules were identified in the fluorescence images and their fluorescence lifetime was determined (Figure S1b). From the reference measurements on glass (τ_{gl}), the mean values of the fluorescence lifetime of both dyes were obtained:

$$\overline{\tau_{gl,542}} = 3.4 \pm 0.1 \ ns$$

$$\overline{\tau_{gl,647N}} = 4.3 \pm 0.1 \, ns$$

These values were used to further calculate the energy transfer efficiency η to graphene for each dye molecule attached to DNA origami structure immobilized on graphene (τ_{gr}):

$$\eta = 1 - \frac{\tau_{gr}}{\overline{\tau_{gl}}}$$

The energy transfer efficiency from an emitter to graphene scales with d^{-4}, where d is a distance between both:

$$\eta = \frac{1}{1 + \left(\frac{d}{d_0}\right)^4}$$

d_0 is the distance of the 50% energy transfer efficiency to graphene, which equals to 17.7 nm and 18.5 nm for a dye ATTO542 and ATTO647N, respectively.[7]

Based on both equations for the energy transfer efficiency, expressed either by the fluorescence lifetime or by the distance d_0, the distance between the dye molecule and graphene can be calculated:

$$d = d_0 \sqrt[4]{\frac{1}{\eta} - 1}$$

The results from Figure S1, expressed by the height at which a dye has been positioned above graphene is presented in Figure 1.

The data was analyzed via a home-written LabVIEW software. The fluorescence lifetime was deconvoluted with FluoFit software from Picoquant.

3.2. Dynamics with GET.

Data acquisition was realized with the home built confocal microscope II (SI chapter 2.1). A 532 nm pulsed laser was used for excitation at 80 MHz repetition rate and an excitation power of 3 µW. The fluorescence was separated into two channels via a 50:50 non-polarizing beam splitter cube followed by subsequent detection by two avalanche photodiodes. This Hanbury-Brown-Twiss detection configuration allows to overcome temporal resolution limits (~ 1 µs) posed by detector dead times and after pulsing. To stabilize Cy3B, a combination of ROXS and oxygen scavenging system is used. The first buffer contains aqueous solution of aged Trolox with PCA (PCA/Trolox12) and the second a 50× PCD (for measurements both buffers were mixed in a 1:50 ratio (50× PCD : Trolox/PCA12).[13,14]

The data was analyzed with a home written Matlab routine based on the photon arrival time correlation algorithm proposed by Laurence et al.[15,16] Technically, we calculate the cross-correlation function as two different signals are correlated. Apart from the following brief description, we denote the correlation as the autocorrelation as the fluorescence coming from the same molecule is split by a 50:50 nonpolarizing beam splitter and detected by two avalanche photo detectors. The cross-correlation of discrete photon arrival time stamps t_i (arrival time of photon i in channel A) and u_j (arrival time of photon j in channel B) is defined as:

$$\hat{C}_{AB}(\tau) = \frac{n(\{(i,j) \ni t_i = u_j - \tau_l\})(T - \tau_l)}{n(\{i \ni t_i \leq T - \tau_l\})n(\{j \ni u_j \geq \tau_l\})}$$

In this equation τ_l represents the lag-time and T the duration of the experiment while $n(\{(i,j) \ni t_i = u_j - \tau_l\})$ counts the number of photon pairs in the time range $(T - \tau_l)$ which fulfill the condition $t_i = u_j - \tau_l$, $n(\{i \ni t_i \leq T - \tau_l\})$ counts the number of photons from channel A which fulfill $t_i \leq T - \tau_l$ and $n(\{j \ni u_j \geq \tau_l\})$ counts the number of photons from channel B which fulfill $u_j \geq \tau_l$.

It is commonly difficult to assign fast correlation components to physical processes as they can be masked by other processes including rotational diffusion and photophysics (e.g. triplet state formation). This problem also arises for our 44 nt tether which exhibits two components in the same time range in the autocorrelation function. With GET, the intensity fluctuation is, however, directly correlated to a change of fluorescence lifetime making it possible to disentangle fluorescence lifetime correlated components from other components by using different subsets of photons for calculating the cross-correlation function. As energy transfer to graphene reduces the fluorescence lifetime, the observed intensity fluctuations are mostly related to photons emitted later after the excitation pulse (i.e. those with higher microtimes, see section B in Figure S2a).

For this time-gated autocorrelation, photon counts in both channels with micro-time stamps according to a previously defined time-gate were neglected prior to calculating the cross-correlation function. The result of such a gating approach is illustrated in Figure S2 where a comparison of different time gates is shown for the pointer-like model system presented in Figure 2 exhibiting 7 nt binding strands. The applied short (section A in Figure S2a, 1 to 2.5 ns) and long (section B in Figure S2a, 2.5 to 12 ns) time gates are illustrated in Figure S2a. The depicted transient shows upper and lower binding events with dwell times in the 100 ms time range. The aforementioned cross-correlation function was calculated for all photons (b, c), for the short time-gate A (d, e) and for the long time-gate B (f, g). It is clearly visible, that the applied time-gates reduce (d, e) or increase (f, g) the contrast of the two observed intensity levels and thus also the resulting amplitude of the cross-correlation function. This enables us to discriminate on-off blinking processes which do not feature changes in intensities due to changes of the fluorescence lifetime but are exclusively related to off-states such as triplet-states, redox blinking or cis-trans isomerization. Applying time-gating to such blinking transients would not influence the amplitude of the cross-correlation amplitude.

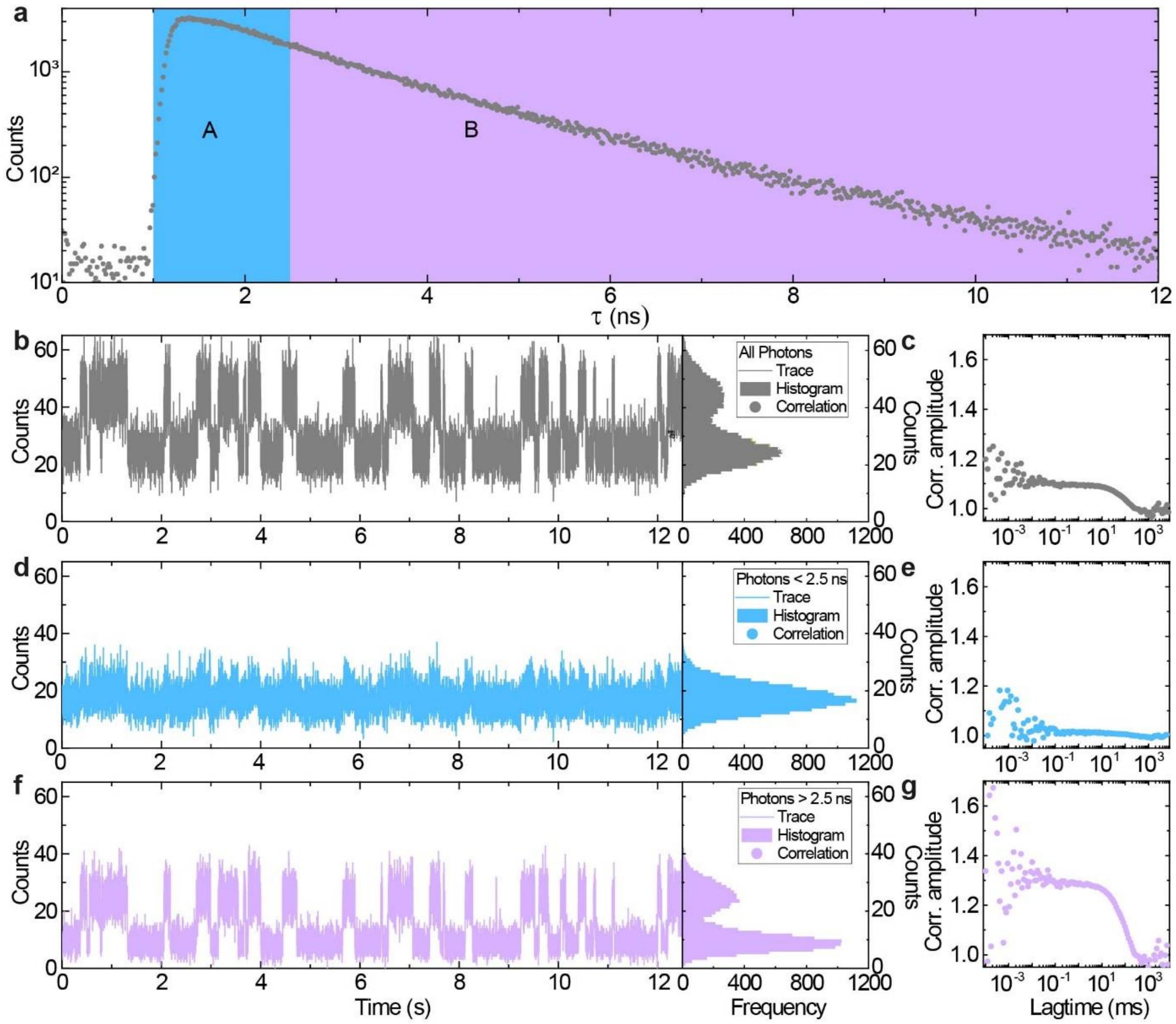

Figure S2. Dynamics with GET. a) Fluorescence decay curve of a DNA origami structure featuring a pointer with Cy3B binding to upper and lower binding strands with 7 nucleotides. The sections A (lilac) and B (blue) illustrate the short (1 to 2.5 ns) and the long (2.5 to 12 ns) time-gate. b) Example transient belonging to the fluorescence decay in (a). c) Correlation function resulting from correlating all photons of the two detection channels with each other. d) Same data as in (b) but with photons belonging to the short time-gate A. e) Correlation function resulting from correlating the photons given in (d). f) Same data as in (b) but with photons belonging to the long time-gate B. g) Correlation function resulting from correlating the photons given in (f).

By comparing cross correlations of "early photons" (short time-gate A, blue graphs in Figure S3), with cross correlations of "late photons" (long time-gate B, lilac graphs in Figure S3), one can distinguish components that are correlated with lifetime changes from those that are not. Accordingly, we assign the short component of the cross correlation shown in Figure S3 (i) to tether fluctuations. Subtraction of the photophysical component leads to a cleared correlation function (Figure S3 ii), that yields a characteristic correlation time of 1.0 ± 0.1 μs. This is in agreement with the expected time scales according to the following simplified model, where the confined diffusion of a flexible tether is approximated by calculating the diffusion coefficient of a 44 bp long dsDNA strand in water to be D = 32 μm^2/s.[17] Using the

characteristic length scale on which the strongest change in fluorescence intensity occurs, approximated with $d_0 = 18$ nm, we calculate the correlation time $\tau_C = \frac{d_0^2}{4D} = 3.1$ µs. The estimated time might be slightly longer due to additional fluctuations of the flexible tether and the spherical distribution of accessible states around the point of attachment. This fast correlation time is sensitive to changes of the diffusion properties of the tether and can be used to detect binding events (streptavidin to biotin at the end of the tether in Figure S3, iii) as well as viscosity changes (buffer with 30% glycerol in Figure S3, iv) as indicated for the cross correlations of exemplary single molecule transients. For the averaged data set in Figure 2e, only transients yielding a cross correlation which could be fitted by a single component in the time range below 5 µs and with negligible further components in the time range above 5 µs were considered.

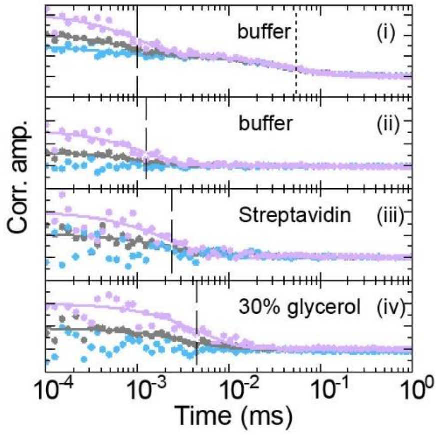

Figure S3. Dynamics with GET. Correlation functions for single transients calculated from all photons (gray), photons belonging to the long time-gate A (lilac) and short time-gate B (blue). (i) shows a raw correlation function of the tether fluctuations in buffer including a longer, photo physical time component. The mono-exponential fit of the short-gated data (blue) in (i) was subtracted to yield the data in (ii). The same correction was applied to the examples for a tether with bound streptavidin (iii) and for the tether fluctuating in buffer with additional 30% of glycerol (iv).

Reference FCS measurements

For estimating the relative change of the correlation time of the tether motion upon binding of streptavidin or changing the buffer conditions to 30% glycerol, we assume a similar effect for the confined diffusion of the tether bound to the L-shaped DNA origami structure as for the free diffusion of the tether without the L-shaped DNA origami structure in solution. Therefore, the tether was folded in absence of the DNA origami structure. The single stranded tether component with the Cy3B dye (1 µl of a 100 µM solution) was mixed with a two-fold access of the single-stranded tether component with biotin (2 µl of a 100 µM solution) to avoid unhybridized fluorescently labeled ssDNA and then folded at 37 °C for 1 h in FOB12.5 buffer. The resulting stock solution was diluted in a ratio of 1:20 with 2M NaCl buffer and then

incubated with 1 µl of streptavidin solution (1 mg/mL). Both solutions were further diluted in a ratio of 1:50 with a combination of ROXS and oxygen scavenging system. The first buffer contains aqueous solution of aged Trolox with PCA (PCA/Trolox12) and the second a 50 × PCD (for measurements both buffers were diluted in a 1:50 ratio (50× PCD:Trolox/PCA12). Afterwards, FCS measurements were performed for both solutions and the resulting normalized cross correlation functions are depicted in Figure S4a. Both curves show negligible components for triplet blinking in the time range between 1 to 50 µs. More important, the binding of streptavidin to the biotin recognition unit can be seen from fitting a 3D diffusion component. Fitting of the data reveals a correlation time (260 ± 10) µs for the pure tether diffusion and (330 ± 10) µs for the tether diffusion with bound streptavidin which gives an increase of the correlation time of a factor of 1.27 due to the increase in hydrodynamic radius.

The changes in correlation time upon increase of the buffer viscosity were measured in a similar way. Therefore, the diffusion was compared via FCS in a combination of ROXS and oxygen scavenging system. The first buffer containing aqueous solution of aged Trolox with PCA (PCA/Trolox12) and the second a 50× PCD (for measurements both buffers were diluted in a 1:50 ratio (50× PCD:Trolox/PCA12) and in buffer with additional 30% of glycerol. The corresponding normalized cross correlation curves are shown in Figure S4b and clearly reveal a reduced diffusion coefficient and thus an increased correlation time for the increased viscosity. While the diffusion in buffer agrees with the data in Figure S4a (280 ± 40 µs), the correlation time in buffer with glycerol increases by a factor of 2.4 to 680 ± 30 µs as revealed by fitting with a 3D diffusion model.

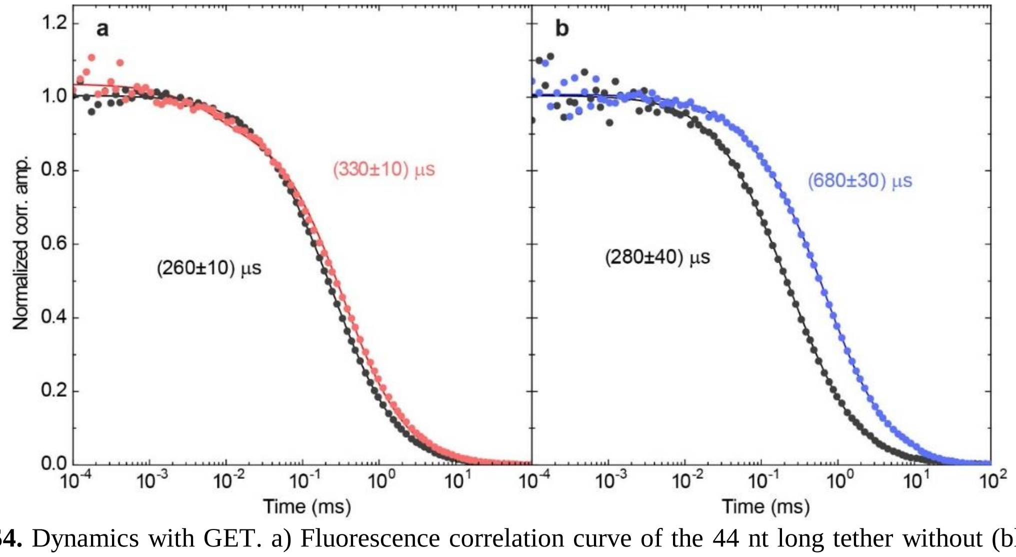

Figure S4. Dynamics with GET. a) Fluorescence correlation curve of the 44 nt long tether without (black) and with (red) incubation with streptavidin. The curves were fitted with a 3D-diffusion component and an additional triplet component. b) Fluorescence correlation curve of the 44 nt long tether in buffer (black) and in buffer with additional 30% of glycerol (blue), curve fitting in analogy to (a).

3.3. Expanding FRET.

The whole data from the FRET experiments were measured on the confocal setup II (SI chapter 2.1) with the acceptor bleaching approach. In case of the static FRET sample, the 532 nm and 640 nm laser powers were set to 1 µW and 9 µW, respectively. For the dynamic FRET sample, the 532 nm laser power was switched to 0.5 µW. The DNA origami stock solution was diluted to a concentration of 25 pM with FOB12.5. To stabilize the FRET pair (ATTO542/ATTO647N) a combination of ROXS and oxygen scavenging system was used.[13,14] The first buffer contains aqueous solution of aged Trolox with PCA (PCA/Trolox12) and the second a 50× PCD (for measurements, both buffers were diluted in a 1:50 ratio (50× PCD:Trolox/PCA12). To verify the correct orientation of the L-shaped DNA-origami structure on graphene, only transients with modulation before and after acceptor bleaching were considered.

The data was analyzed via a home-written LabVIEW software. The fluorescence lifetime was deconvoluted with FluoFit software from Picoquant.

The FRET efficiency E and the FRET rate k_{ET} are calculated with the fluorescence lifetime of the donor in presence of the acceptor τ_{DA} and after acceptor bleaching τ_D.

$$E = 1 - \frac{\tau_{DA}}{\tau_D}$$

$$k_{ET} = \frac{1}{\tau_{DA}} - \frac{1}{\tau_D}$$

Based on E and including the distance r_0 where 50% of the energy is transferred to the acceptor (for ATTO542/ATTO647N r_0 = 6.32 nm), the distance between donor and acceptor is calculated. The FRET distance is calculated from data measured on glass to avoid influence of a potentially slightly changed energy transfer rate constant on graphene.[18–21]

$$r = \sqrt[6]{\frac{1}{E_{gl}} - 1}\, r_0$$

The GET efficiency η and GET rate constant k_G are calculated with the fluorescence lifetime of species on glass (index gl) and graphene (index gr). A line above results indicates an average from previous calculations (exemplary shown for the acceptor species):

$$\eta_A = 1 - \frac{\tau_{A,gr}}{\overline{\tau_{A,gl}}}$$

$$k_{G,A} = \frac{1}{\tau_{A,gr}} - \frac{1}{\overline{\tau_{A,gl}}}$$

The GET distance is calculated from η and the distance d_0 from the dye to graphene where 50% energy transfer is observed (17.7 nm for ATTO542 and 18.5 nm for ATTO647N).[7] For these calculations, only the acceptor and the donor after acceptor bleaching are taken into account because the donor in presence of the acceptor shows a lower η compared to the donor after acceptor bleaching due to the additional FRET rate constant k_{ET}, (exemplary shown for the donor):

$$d = \sqrt[4]{\frac{1}{\eta_D} - 1}\, d_{0,D}$$

Finally, the angle between donor and acceptor δ was extracted.

$$\delta = \mathrm{acos}\left(\frac{a-d}{\bar{r}}\right)$$

When the direction of FRET is close to vertical, e.g., for the vertical structure of the static FRET experiment (Figure 3f) and for the "up" binding mode of the dynamic FRET experiment (Figure 4f), the numerator can be bigger than the mean distance $\bar{r}$ (the denominator). In this case, δ is not defined and the angle is set to 0° or 180°, respectively (also see bars at 0° for Figure 3f and 4f). To obtain meaningful mean angles for the underlying populations, we calculated the mean angles from the mean distances to the surface: $\bar{\delta} = \mathrm{acos}\left(\frac{\overline{a-d}}{\bar{r}}\right)$.

The evaluated data is illustrated in Figure S5. The fluorescence lifetime of the acceptor on glass (red line, 4.0 ns) and graphene (orange line, 1.2 ns) for every subspecies (h, d, v) is the same indicating that the distance to graphene is the same (Figure S5a). Whereas the same behavior is observed by the donor after acceptor bleaching on glass (turquoise line, 3.0 ns), the donor after acceptor bleaching on graphene is varying because of the different distance of the donor to graphene. The donor lifetime in presence of the acceptor on glass shows the influence of FRET and the same sample on graphene has an additional GET contribution.

The FRET efficiency E decreases from horizontal over diagonal to vertical on glass as well as on graphene (Figure S5b,d). In addition, the FRET efficiency also decreases on graphene in comparison to glass. The FRET rate constant k_{ET} (d) is not affected by the graphene in the diagonal and vertical case but in the horizontal case k_{ET} is slightly increasing on graphene compared to the glass sample. This might indicate an enhanced energy transfer from the donor to the acceptor mediated by graphene which has been postulated in theoretical works.[18–21]

The GET data (Figure S5c, e) also shows the influence of FRET. In the presence of the acceptor (DA), the donor is always shifted to lower GET efficiencies η than after acceptor bleaching (D).

This is caused by the competing FRET rate constant k_{ET}. The GET efficiency and the rate constant k_G stay constant for every acceptor as expected (Figure S4c,e).
The calculated FRET distance (Figure S5f) for the glass sample shows a decrease from horizontal over diagonal to vertical which is already observed in the fluorescence lifetime and FRET data. While the acceptor GET distance is constant, the donor distance *d* to graphene varies as designed (Figure S5g). From the combined information of Figures S4f and g the angle δ is calculated. The horizontal sample has an angle of 87.0 ± 0.6° which is close to the designed angle of 90°. The angle for the diagonal (ex: 27.9 ± 0.6°, designed: 37°) and vertical samples (3.7 ± 14.0°, designed: 0°) agree well with the designed angles.
In conclusion, the combination of FRET and GET shows a novel approach to determine the position of a donor-acceptor pair in space.

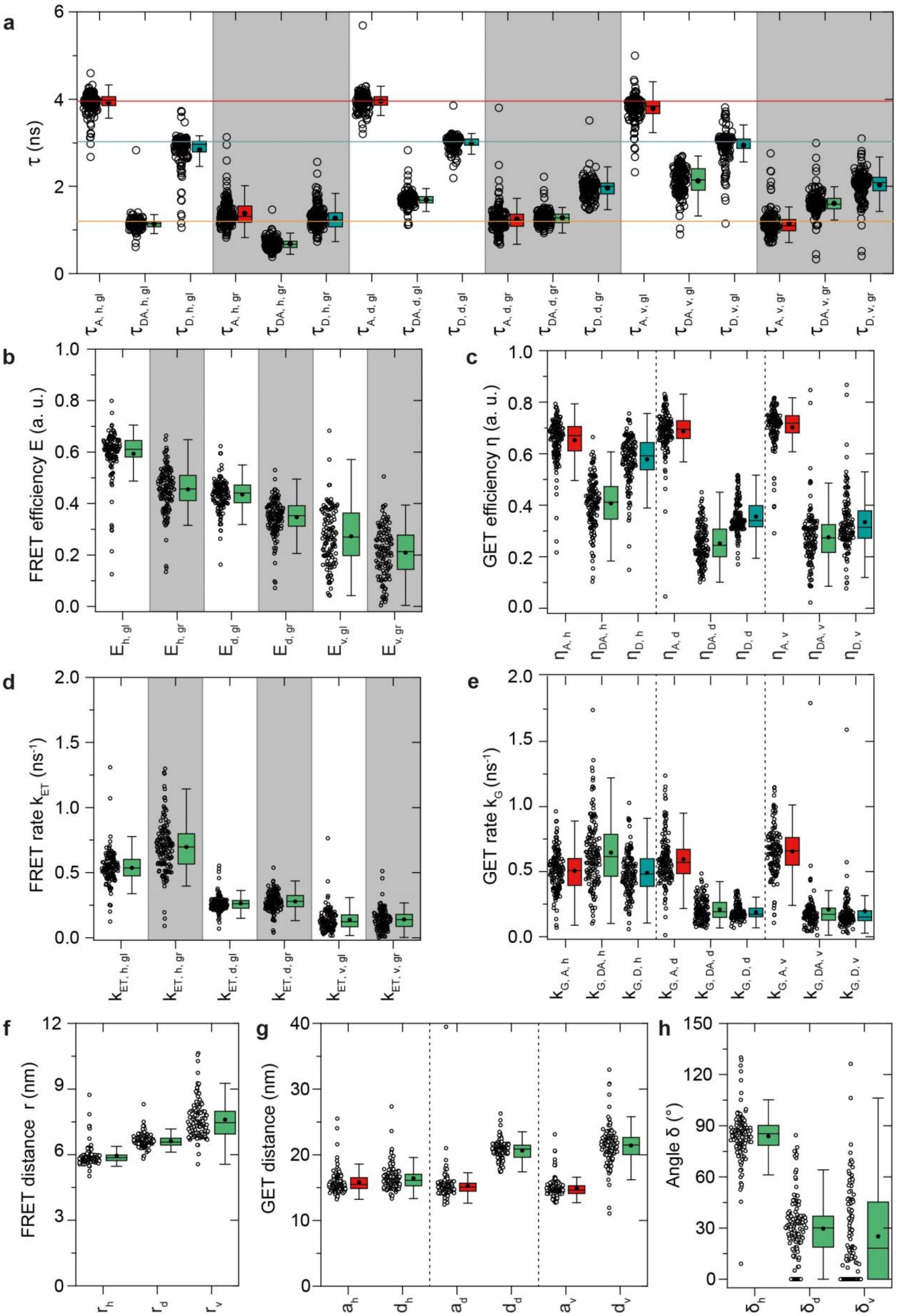

Figure S5. Measured and calculated data of the static FRET experiments. The data is illustrated by hollow dots (∘) and a box plot. In the box plot, the dot (•) indicates the mean average, while the line (-) is the median. The

percentile ranging from 25% to 75% and the whiskers are in the range of 1.5 IQR. A gray background implies a measurement on graphene. a) shows the fluorescence lifetime of all species with average fluorescence lifetime of the acceptor on glass (red line, $\overline{\tau_{A,gl}}$ =4.0 ns), donor after acceptor bleaching (turquoise line, $\overline{\tau_{D,gl}}$ = 3.0 ns) and acceptor on glass (orange line, $\overline{\tau_{A,gr}}$ = 1.2 ns). Overall a quenching in presence of FRET and/or GET is observed. On the one hand, the FRET efficiencies on graphene are lower compared to the FRET efficiency of the same species on glass due to the additional graphene rate constant (b)). On the other hand, the FRET rate constant is similar besides the horizontal case where the FRET rate constant is slightly increased in presence of graphene (d)). The GET efficiencies are similar for all acceptor species and increase for the donor with smaller distance to graphene and FRET (c)). The GET rate constant is not influenced by the presence of FRET (e)). Based on E and with a r_0 of 6.32 nm for ATTO542 and ATTO647N, the FRET distance was calculated and is shown in f). The illustrated GET distances (g)) were calculated with a d_0 of 17.7 nm for ATTO542 and 18.5 nm for ATTO647N. h) Finally, the angle was calculated and shows the expected tendency of lower angle for the different species.

For the dynamic FRET samples, information about the conformational dynamics is additionally obtained. The dynamics can be extracted by the fluctuation of FRET (on glass, Figure 4b), GET (on graphene, after acceptor bleaching Figure 4c) or both (on graphene in presence of the acceptor, Figure 4c). The sample on glass only shows a modulation in presence of the acceptor (until 5 s), after acceptor bleaching the modulation disappears. On the graphene sample, modulation in presence and absence of the acceptor is observed. During the first excitation with green (until 6 s) the modulation is caused by the combined influence of FRET and GET, after the acceptor bleached the modulation is caused only by GET.

The “on” time, t_{on} (binding time) is extracted from autocorrelation analysis and the modulation M is calculated from the fluorescence lifetime of the “up” and “down” position.

$$M = \frac{\tau_{up} - \tau_{down}}{\tau_{up} + \tau_{down}}$$

The analysis of the dynamic FRET experiment is summarized in Figure S6. The fluorescence lifetime is decreasing in the presence of GET and FRET (Figure S6a). The conformational dynamics of the pointer are not influenced by GET and/or FRET, and the on time (t_{on}) for “up” binding event is always 80 ms, while a “down” binding event has 150 ms (Figure S6b). As expected, the modulation is the smallest for FRET only ($M_{DA,\,gl}$), and is increasing from GET only ($M_{D,\,gr}$) to the combination of both ($M_{DA,\,gr}$) (Figure S6c).

The FRET data (d, f) shows a similar behavior as the static FRET samples. The FRET efficiency is lower on graphene which is caused by the additional GET rate constant k_G. Interestingly, the FRET rate constant k_{ET} is increasing on graphene for the binding closer to graphene while k_{ET} for the upper binding site is the same on glass and on graphene.

The GET efficiency η (Figure S6e) is increasing after the acceptor bleaches as the competing FRET process disappears. η gives an idea of the relative orientation of the acceptor to both binding sites of the flexible donor strand. The acceptor is closer to the lower binding site than to the upper binding site. The GET rate constant k_G (Figure S6g) is smaller for the upper than for the lower binding site with the acceptor exhibiting an intermediate value.

The FRET distance (Figure S6h) which is calculated from the glass dataset shows a narrow distribution. The GET distance (Figure S6i) gives a first impression of the relative orientation of acceptor and both donor binding modes. Only the angle δ yields the whole information about orientation between donor and acceptor molecule (Figure S6j). For the “up” binding, the δ distribution shows a peak at 0°, as we set all data points for which the dye distance determined by GET is larger than the distance determined by FRET to zero (see discussion for static GET-FRET). Using the averages of the FRET distance and the GET values, we determine an average angle of $\delta = 19.0 \pm 6.9°$. An angle of $111.5 \pm 2.17°$ for the lower position indicates that the donor is lower than the acceptor, because the angle is above 90°.

To sum up, the combination of FRET with GET is not only limited to static systems but can also be expanded for dynamic systems to verify the orientation of a donor and acceptor in space. Importantly, the dynamics are not influenced by GET.

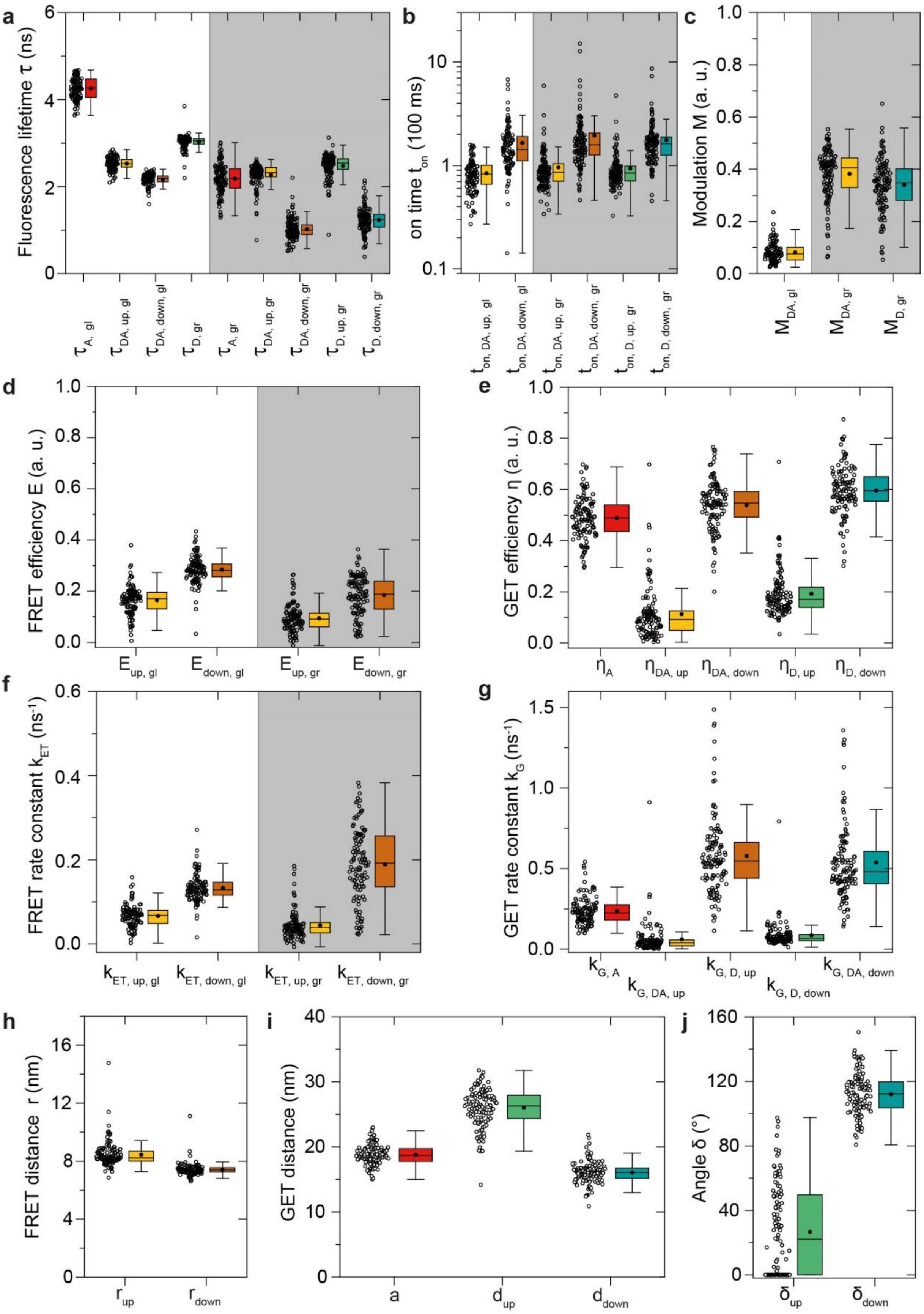

Figure S6. Measured and calculated data of the dynamic FRET experiments. The data is illustrated by hollow dots (∘) and a box plot. In the box plot the dot (•) indicates the mean average, while the line (-) is the median. The percentile ranging from 25% to 75% and the whiskers are in the range of 1.5 IQR. A gray background implies a measurement on graphene. The influence of graphene, FRET and subspecies (up and down) to the fluorescence

lifetime is illustrated in a). The on times for the “up” and “down” binding are independent of FRET and GET as expected (b)). c) shows the modulation which is higher in present of FRET and GET ($M_{DA, gr}$) followed by the Get only sample ($M_{D, gr}$) and FRET only sample ($M_{DA, gl}$), The FRET efficiencies are lower on graphene compared to glass due to the additional GET rate constant (d)). The FRET rate constant increased slightly closer to the graphene surface (f)). The competing FRET process decreases the GET efficiencies (e)), while the GET rate constants remained constant (g)). Based on the FRET efficiency and including the r_0 of 6.32 nm for ATTO542 and ATTO647N the FRET distance was calculated and is shown in f). The illustrated GET distances (g)) were calculated with a d_0 of 17.7 nm for ATTO542 and 18.5 nm for ATTO647N. Finally, based on h) and i) the angle for the “up” and “down” position was extracted (j).

3.4. Graphene biosensing.

A Secure Seal™ hybridization chamber was first glued on a glass coverslip in a way that the entire surface of previously transferred graphene was fully covered. The concentration of the pillar-shaped DNA origami labeled with pyrene-modified oligos was adjusted to 50 pM in 1× FOB12.5. Then the chamber was filled with 150 µL of the diluted sample, and after 1-2 minutes, the chamber was washed thrice with 1× FOB12.5 buffer. The sample was measured to collect fluorescence lifetime and intensity information for the free-capture strand and capture strand hybridized with a closing strand. Afterwards, the chamber was filled with 150 µL of 5 µM ssDNA (GTGGTATTCGAAAACAAAATCACCATCAATAACCCTCAATAAAT) and incubated for 30 minutes at room temperature, for graphene surface passivation. This was followed by the incubation with a target strand (38 nucleotides at 5 nM concentration) for 3 hours at 37 ˚C. Afterwards, the chamber was washed thrice with 1× FOB12.5 buffer. The sample was measured again to collect fluorescence lifetime and intensity information for the capturing strand hybridized with a target.

For these experiments, three types of the pillar-shaped DNA origami structures with the hairpin-like assay were prepared: 1) a structure with a freely moving capture strand (39 nucleotides long) with an attached ATTO643 at the height of 16.3 nm, 2) same as (1) with an additional closing strand at 9.2 nm, to which a capture strand may hybridize (getting into closed form of the assay), 3) like (2) with additional biasing strand incorporated at 30.6 nm to catch and stabilize the capture strand after the target detection (open form of the assay). Each type of the nanostructure (sketches depicted in Figure 5 and S7) was measured before and after the incubation with a target, such that six forms in total could be imaged in order to get insight into the influence of all the components on the performance of the bioassay.

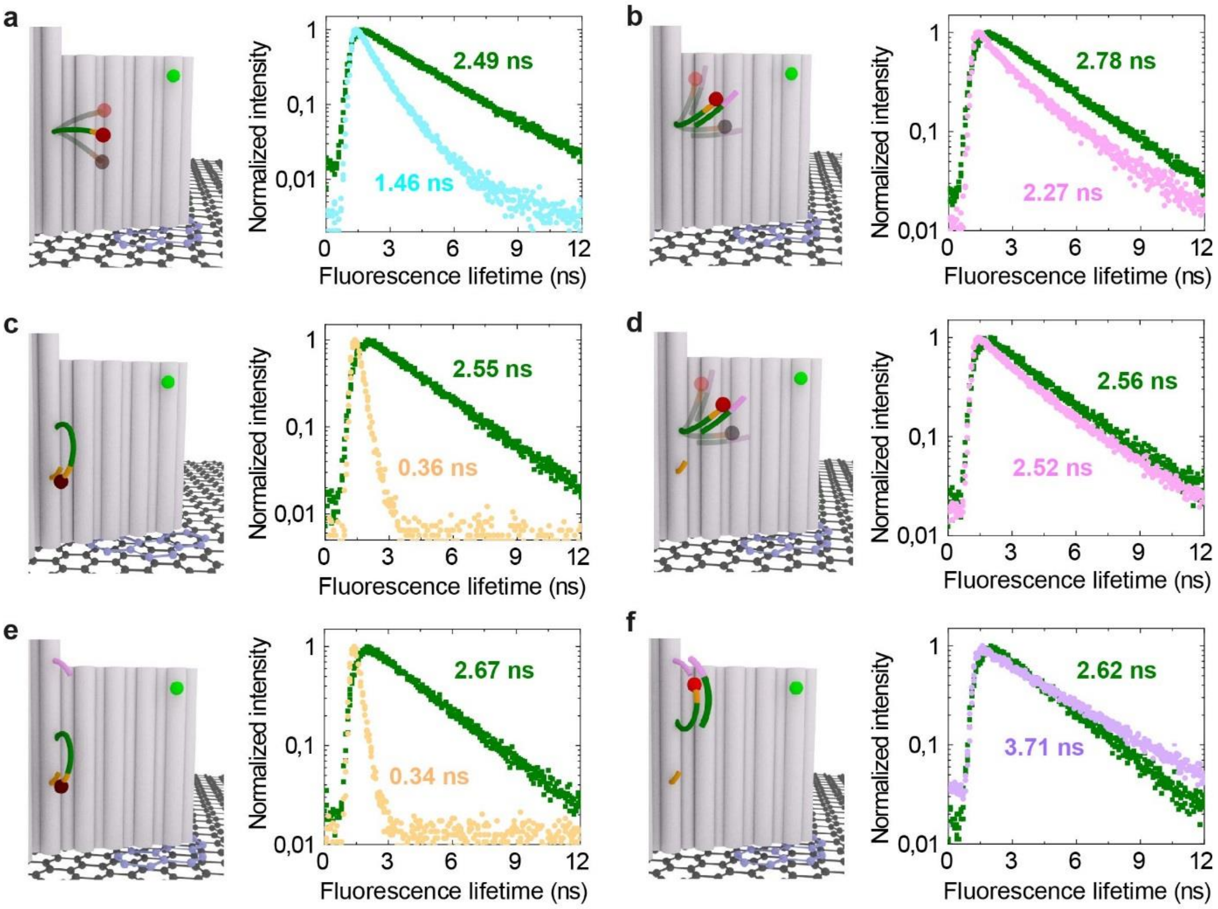

Figure S7. Graphene biosensing with a nucleic acid bioassay. Sketches (left panels) and fluorescence decays with fluorescence lifetime values obtained from the monoexponential fit (right panels) of an ATTO542 dye (internal reference) and an ATTO643 dye attached to the capture strand which is: (a) free to move (pale cyan), (b) bound to a target strand (pale magenta), (c and e) hybridized with a closing strand (orange), (d) liberated by a target strand (pale magenta), (f) liberated by a target strand and additionally caught and stabilized by a biasing strand (lilac). In all cases an ATTO542 dye was used as an internal reference, to monitor the proper orientation of a DNA origami structure on graphene and high quality of graphene.

Each pillar-shaped DNA origami structure was additionally labeled with an ATTO542 dye at a height of 23.4 nm from graphene, serving as an internal reference. It was used as an indicator of the quality of graphene as well as the functionality of the DNA origami structure itself. All measurements for this part were carried out on the confocal setup I (SI chapter 2.1) with a pulsed interleaved laser excitation of 532 nm and 639 nm. Single molecules were identified in the fluorescence images and their fluorescence lifetime was determined. For further analysis, only the colocalized spots were considered, assuring the presence of the assay with an ATTO643 dye and a reference ATTO542 dye. As an example of the entire population of all the measured colocalized molecules within one assay, we present the results obtained for the pillar-shaped DNA origami structure with a freely moving capture strand in Figure S8. Using the equations from part 3.1 of the SI, we calculated that an ATTO542 at the height of 23.4 nm, should have the fluorescence lifetime of 2.7 ± 0.3 ns. In the upper histogram in Figure S8, in

green we marked the population of the structures assigned as those with properly quenched fluorescence of the reference dye (fluorescence lifetime in the range of 2.4 – 2.9 ns). This indicates properly oriented DNA origami structure on graphene, keeping its full functionality, as well as the high quality and clean graphene. In the scatter plot, these nanostructures were marked with grey squares filled with cyan circles. On the other hand, the presence of all spots out of this range (grey histogram and empty squares in the scatter plot), may result from various deviations, such as tilting of a pillar-shaped DNA origami structure, but also defects or polymer residues on graphene. For example, the population around $\tau_{ATTO643}$ = 4.3 ns and $\tau_{ATTO542}$ = 3.4 ns indicates no fluorescence quenching of both dye molecules, resulting most probably from the presence of holes or defects in graphene or of PMMA residues. This analysis based on the fluorescence lifetime of the reference dye ATTO542 was applied for all the measured samples (final results depicted in Figure 5).

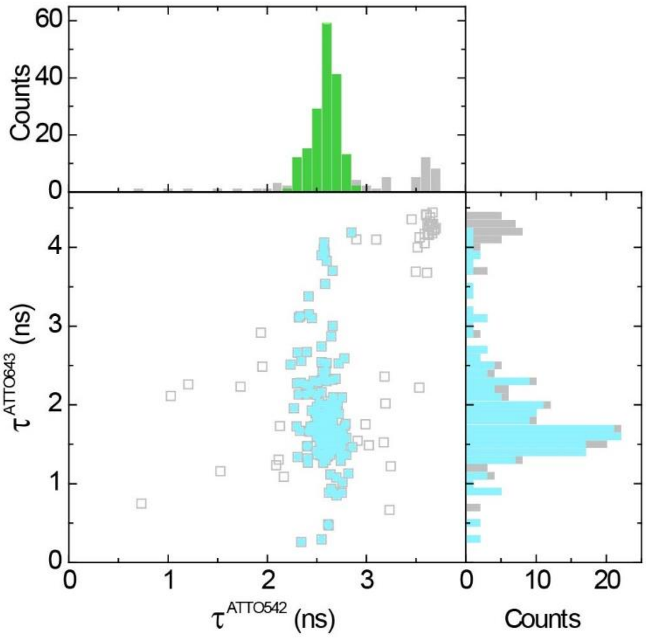

Figure S8. Graphene biosensing with a nucleic acid bioassay. Scatter plot of fluorescence lifetime of colocalized dye molecules (both dye molecules within one pillar-shaped DNA origami structure): ATTO643 at the 3' end of the freely moving capture strand (check sketch in Figure 5a and Figure S6a) and ATTO542 as an internal reference at the fixed height of 23.4 nm. In grey (□) all acquired data points and in cyan (●) population of only properly oriented pillar-shaped DNA origami structures and clean graphene (fluorescence lifetime of ATTO542 in the range between 2.3 and 2.9 ns). In the further analysis for all measured samples, such threshold for the fluorescence lifetime of ATTO542 is applied. In the top panel histogrammed fluorescence lifetime of ATTO542: in grey for all data points, in green for properly oriented DNA origami structure and high-quality graphene samples. In the right panel analogous histogram for an ATTO643 dye at the capture strand: in grey all acquired results, in cyan – data points selected for further analysis.

In order to explain the presence of the population at around 3 ns in the full assay after target binding (gray arrow in Figure 5f), called "free capture strand + target", we checked with a self-written Python code, whether the target can unintentionally bind to the DNA origami structure.

Indeed, it turned out that a fragment of the target (TA**T**AC) can stick to one of the staples in the pillar-shaped DNA origami structure at the height of ~23.1 nm, which exactly matches to the population at around 3 ns. Therefore, we modified the highlighted sequence of the target by one nucleotide (T → A) and performed the measurements again. In Figure S8, we compare both sets of measurements, (a) with the old and (b) with the new target sequence. While in the closed form (a capture strand caught by the closing strand, orange population), a very similar narrow distribution < 0.5 ns is obtained, there is a significant difference for the open form (target hybridized to the capture strand and additionally caught by the biasing strand at about 31 nm distance from graphene, lilac population). The population at about 3 ns is significantly smaller and the distribution of the fluorescence lifetime is narrower. With this small modification of the target molecule, we could further improve the performance of the GET-based bioassay.

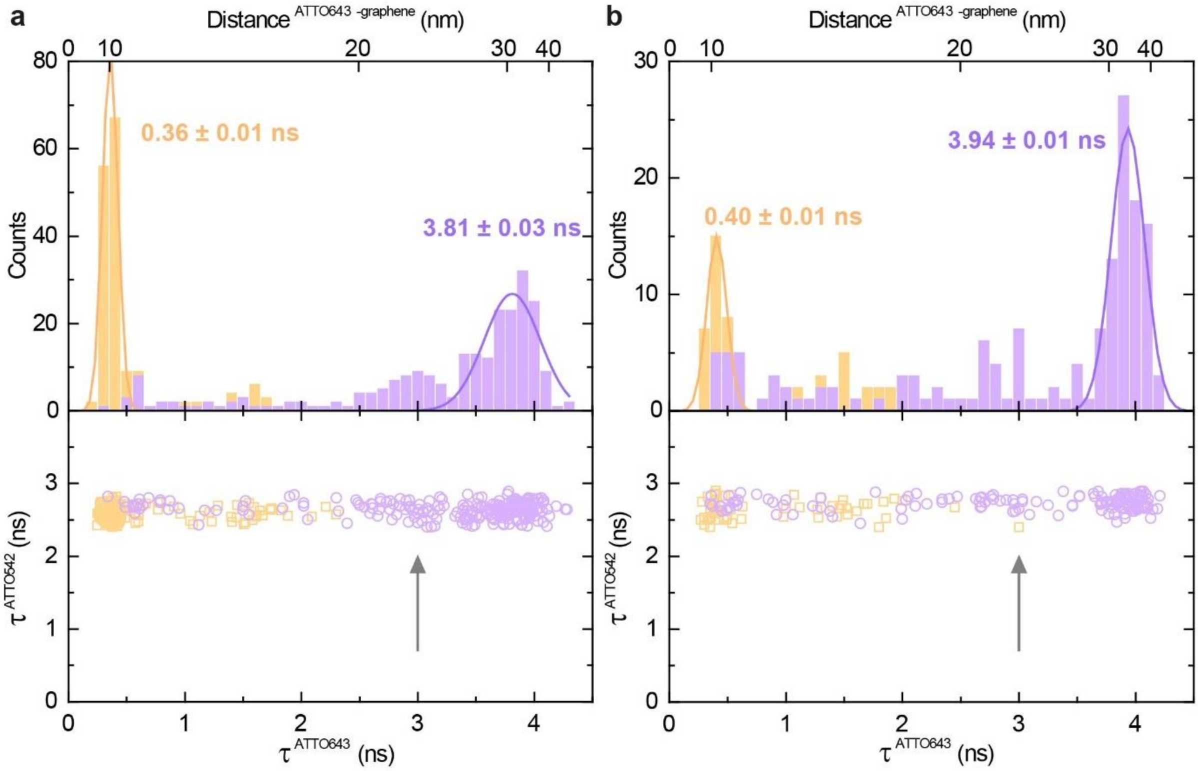

Figure S9. Graphene biosensing with a nucleic acid bioassay. Scatter plots (bottom panels) and corresponding histograms (top panels, fitted with a Gaussian function (the mean value and standard error obtained from the fit)) of fluorescence lifetime of colocalized dye molecules: ATTO643 at the 3' end of the capture strand and ATTO542 as an internal reference at the fixed height of 23.4 nm (check sketches in the Figure 5f and S7e-f). The full assay before (orange) and after (lilac) the detection of (a) the old target and (b) the new target. A significant reduction of the population at around 3 ns (marked with the arrow) is noted after exchanging just one nucleotide (T→A) of the target sequence, thereby eliminating a fragment (TA**T**AC) causing unspecific sticking to one of the staple strands of the DNA origami structure at the height of ~23.12 nm. For more details check Table S8.

3.5. GET tracking.

The data for the tracking experiments was measured on widefield setup I with a 560 nm laser power of 8 mW (200 × 200 pixel, 93 nm/pixel) with an EM-gain of 10, exposure time of 300

ms and an overall time of 400 frames. The DNA-origami structure concentration was adjusted to 25 pM and stabilized with 1:50 50× PCD:PCA/Trolox12.

The superresolution image was generated via Picasso software package, for fitting of the PSF, the MLE (maximal likelihood estimation) analysis was used and further processed with a homewritten LabVIEW software.[22] For the GET tracking analysis, one out of the triple Gaussian distribution (see Figure 6 in the main text) was defined to be at a distance of 24 nm from graphene for referencing, in accordance with the DNA origami nanostructure design.

Examples of the tracking are illustrated in Figure S10 (animated trajectories are illustrated in Supplementary video 1-3). As a reminder, in the x/y projection only two populations should be observed because the "up" and "low" binding site only differ by the z component. The distance to "mid" binding site should be around 5.4 nm. The two populations are most of the time only hard to distinguish in the x/y projection. But the additional GET superresolution shows three defined populations along either y/z or x/z or both.

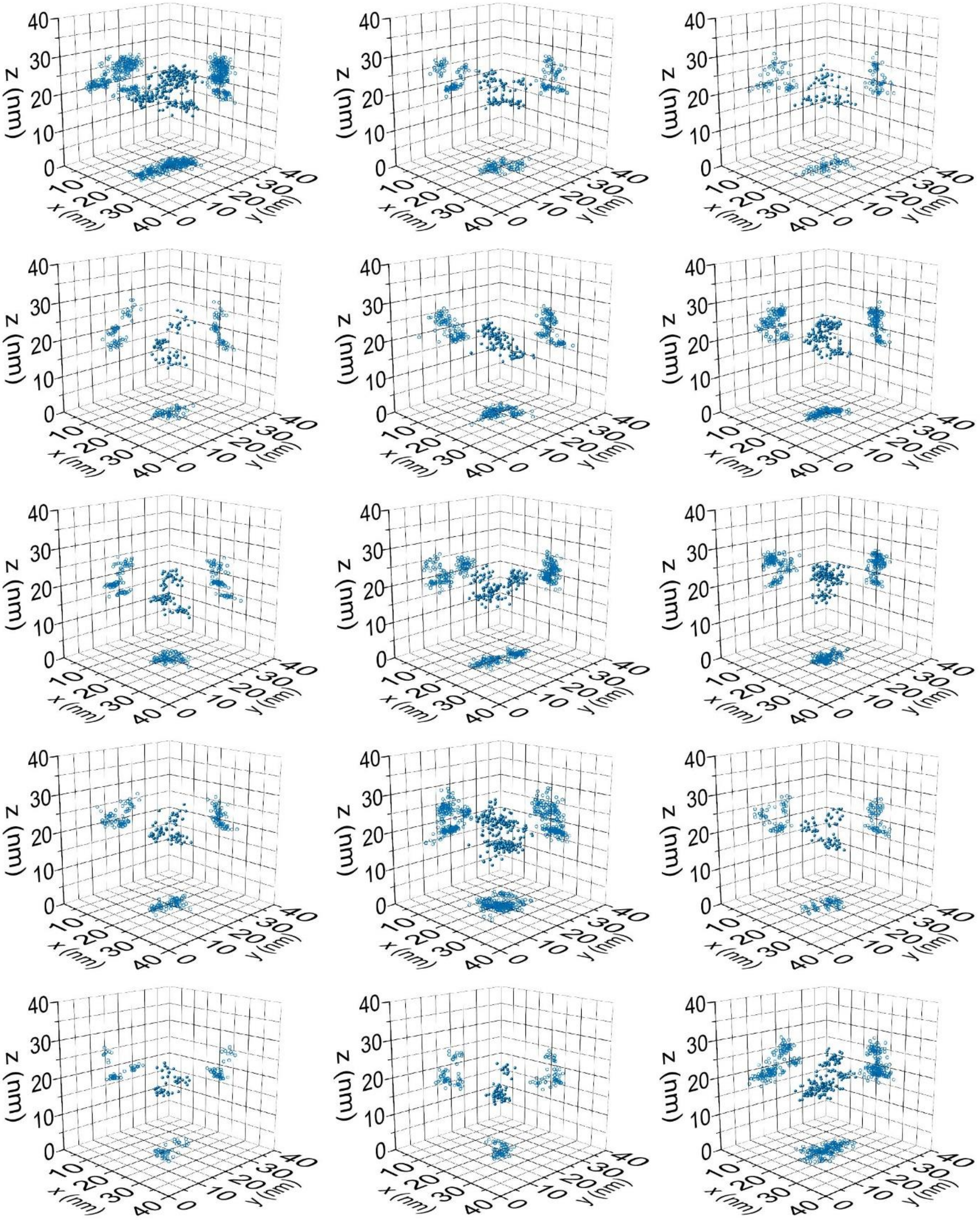

Figure S10. Superresolution tracking images. The blue spheres in the center illustrate the binding events. On the sides the projection along x/y, x/z and y/z are shown. For analyzing, the highest population is always put to 24 nm.

3.6. GET-DNA PAINT superresolution.

The data for the GET-superresolution was measured on widefield setup II (SI chapter 2.1) with a 560 nm laser power of 200 mW (200 × 200 pixel, 100 nm/pixel) with an EM-gain of 10, exposure time of 100 ms and 65000 frames in total. After incubation of the DNA origami

structures with a concentration of 800 pM in FOB12, the graphene surface was passivated to avoid sticking of the paint oligonucleotides. For the passivation, an oligonucleotide with an unspecific sequence (50 µL, 1 µM) was incubated for 1h at room temperature. The sample was measured with 2 nM Imager (ATTO542, 8 nt, sequence: CGGGCATT-ATTO542) in Glox12 buffer.

The superresolution image was generated via Picasso software package, for fitting of the PSF, the MLE (maximal likelihood estimation) analysis was used and further processed with a home-written LabVIEW software.

For the analysis, the average intensity $\bar{I}$ from the whole data was set to the height d = 17.9 nm (average between upper binding site (19.2 nm) and lower binding site (16.5 nm)).

Including d_0 (17.7 nm), the reference intensity I_{ref} (intensity without any energy transfer to the graphene) was calculated.

$$I_{ref} = \frac{\bar{I}}{\left(1 - \frac{1}{1 + \left(\frac{d}{d_0}\right)^4}\right)}$$

Based on this result, the GET distance was calculated from every intensity value.

$$d = d_0 \sqrt[4]{\frac{1}{\left(1 - \frac{I}{I_{ref}}\right)} - 1}$$

Additional superresolved images are illustrated in Figure S11. In every odd row the x/y image is shown and in every even row the x/z projection is shown. The color scale is centered between the lines of the DNA origami cube with positive values shown in green and negative values shown in lilac.

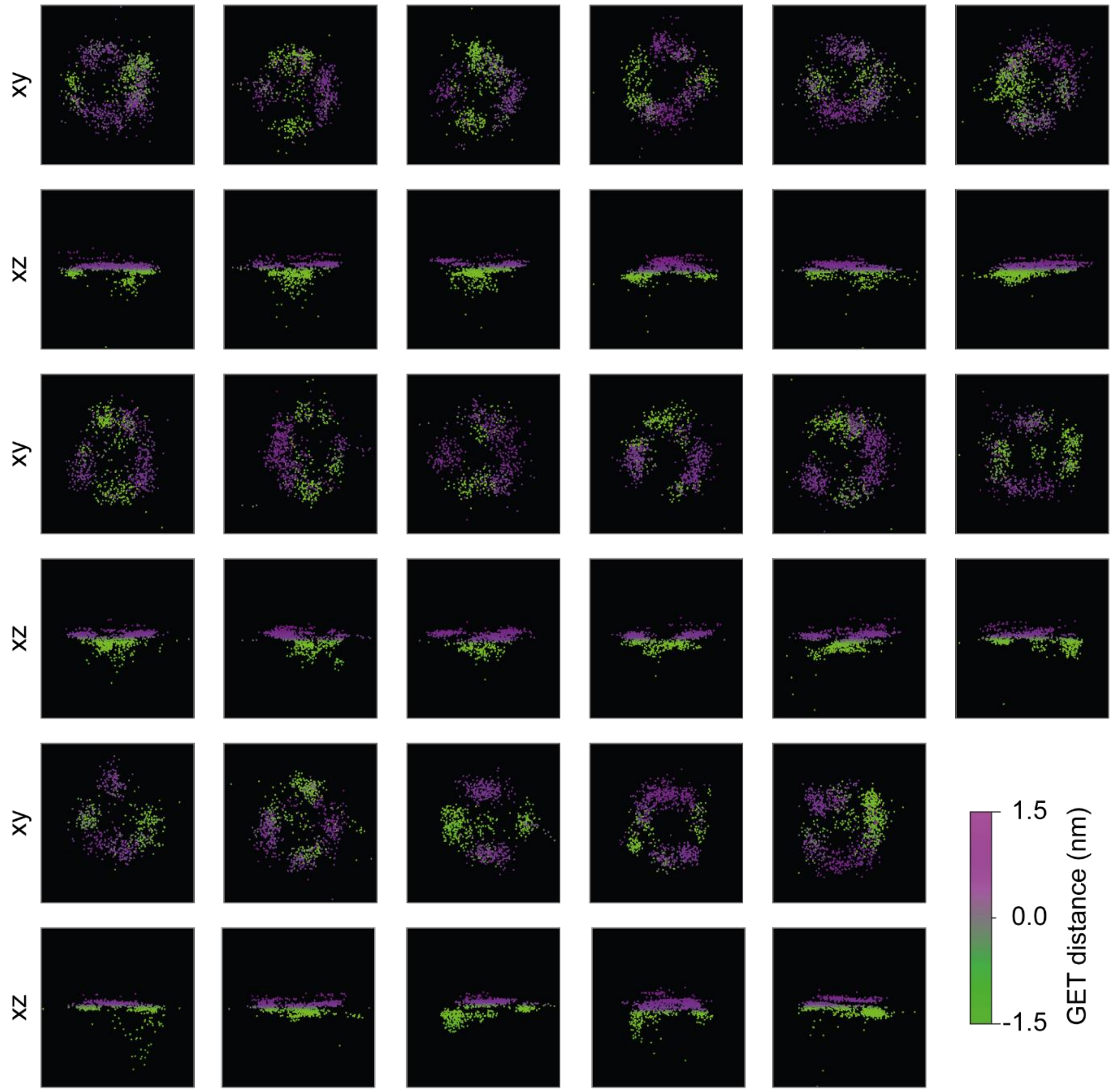

Figure S11. GET-DNA PAINT superresolution. Superresolved 70 × 70 nm images of the DNA origami cube. On the upper row the x/y projection is shown and on the lower, the x/z. The color scale is put to the center of the DNA origami structure binding events below this center are illustrated in green and above are in lilac.

4. DNA origami structure design

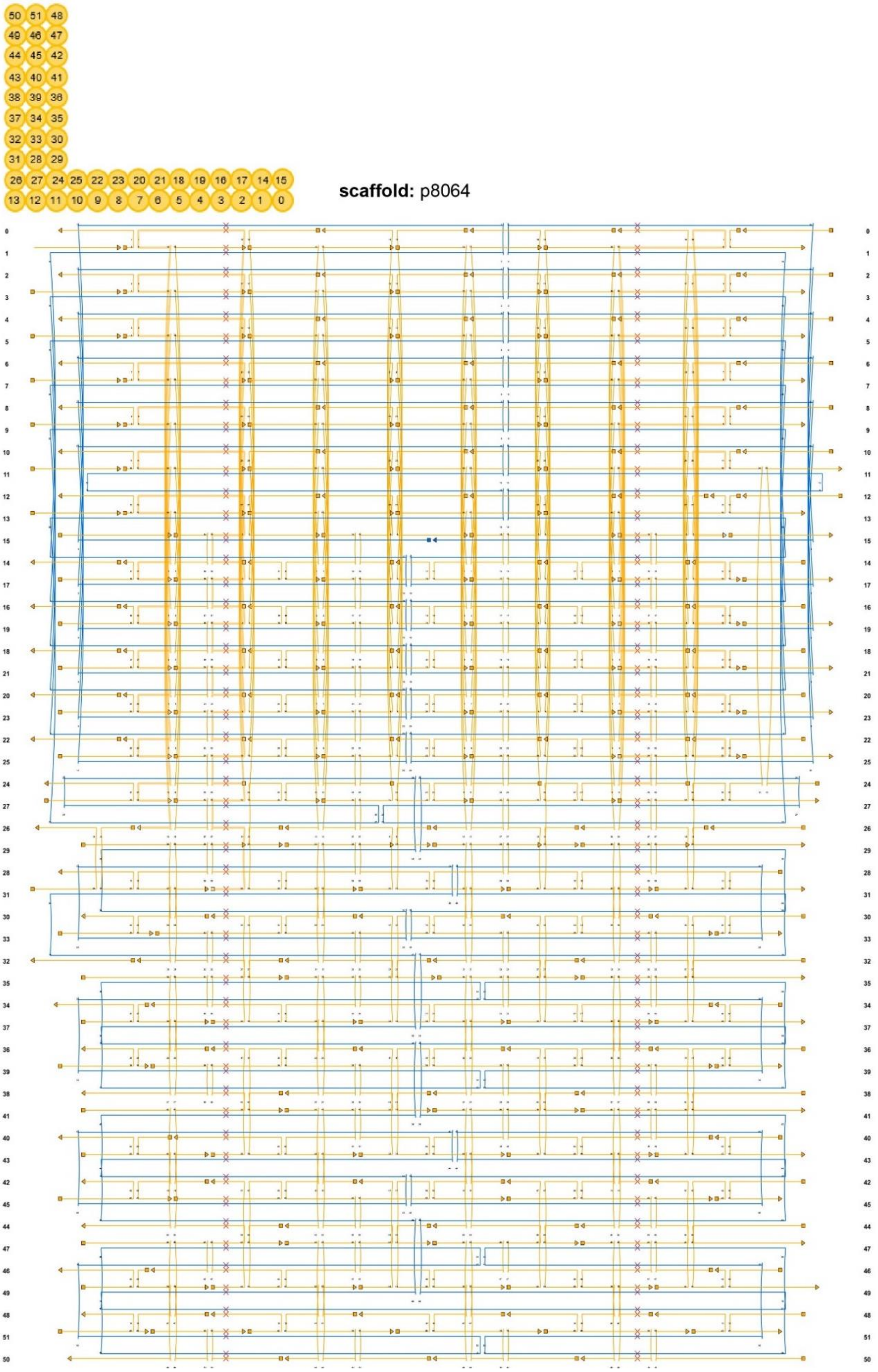

Figure S12. Cadnano design of the L-shaped DNA origami structure. Zoom in to see details.

5. DNA sequences

Table S4. Core staples from the 5’ to the 3’ end for the pillar-shaped DNA origami structure.

Staple ID	Sequence (5’ to 3’)
P1	GAGAAGGCATCTGCAATGGGATAGGTCAAAAC
P2	AACCGTGTCATTGCAACGGTAATATATTTTAAATGAAAGGGT
P3	ATCGGTCAGATGATATTCACAAACCAAAAGA
P4	GCTGGCATAGCCACATTATTC
P5	CTGTATGGGATTACCGTTAGTATCA
P6	CCATAATGCCAGGCTATCAAGGCCGGAGACATCTA
P7	CTCATCGGGATTGAGTGAGCGAGTAACAACCCGTC
P8	TAGCCAGCTTTCATCCAAAAATAAACGT
P9	TAGCCTCAGAGCATACCCTGT
P10	AATACCCCAACATTCATCAAAAATAATTCGCGTCT
P11	GGCTAAAACTTCAGAAAAGTTTTGCGGGAGATAGAACC
P12	CCCGGTTGATAAAGCATGTCAATC
P13	ATCGATGCTGAGAGTCTACAAGGAGAGGGAACGCCAAAAGGA
P14	GACAATTACGCAGAGGCATTTTCGAG
P15	TAAGTTGGCATGATTAAAGAA
P16	CCAATGTTTAAGTACGGTGTCCAAC
P17	CGGAATAGAAAGGAATGCCTTGCTAAACAACTTTCAAC
P18	GAGTTAAAAGGGTAATTGAGCGCTAATATCAGAGGAACTGAACACC
P19	TTTAGCGATACCAACGCGTTA
P20	TTTTTGCGGATGCTCCTAAAATGTTTAGATGAATTTTGCAAAAGAAGTT
P21	AATAAAACGAACTATGACCCCACCAAGC
P22	AATATCGTTAAGAGAGCAAAGCGGATTGTGAAAAATCAGGTCTTT
P23	ATTACGAGATAAATGCCAGCTTTGAGGGGACGACGACAG
P24	ACAACGCCTGTAGCATTTACCGTATAGGAAG
P25	TTACCATTAGCAAGGCCTTGAATTAGAGCCAGCCCGACTTGAGC
P26	CAGCAGCGCCGCTTGTTTATCAGCTTCACGAAAAA
P27	CTTACGGAACAGTCAGGACGTTGGGAAGAAA
P28	AGCTCTTACCGAAGCCCAATA
P29	TATTACGAATAATAAACAAATCAGATATGCGT
P30	CACGGCAACAATCCTGATATACTT
P31	CATCGAGATAACGTCAAACATAAAAGAGCAAAAGAATT
P32	CAAGCCCAATAGGAACCACCCTCACCCGGAA
P33	CATTTCGCAAATGTCATCTGCGAACGAGAGATTCACAATGCC
P34	GGCGCAGACGGTCAATCATCGAGACCTGCTCCATGTGGT
P35	CAAACGGAATAGGAAACCGAGGAATAAGAAATTACAAG
P36	ACCAACAAACCAAAATTAACAATTTCATTTGAATTACCGAGG

Staple ID	Sequence (5’ to 3’)
P37	CATTTGAGATAACCCACGAAACAATG
P38	AGGACAGATGAACGGTGTAACATAAGGGAACCGAAGAAT
P39	TGGCTTTTTACCGTAGAATGGAAAGCG
P40	GTTAAAGGAAAGACAGCATCTGCCTATTTAAGAGGCAGGAGGTTTA
P41	AGTAGGTATATGCGTTATACA
P42	CGAACACCAAATAAAATAGCAGCCAAGTTTGCCTTTAGCGTCAGA
P43	GCGAAACAAAGTGTAAAACACATGGCCTCGATTGAACCA
P44	AAGAAAGCTTGATACCGCCACGCATACAGACCAGGCGCTGAC
P45	CTGAATATAGAACCAAATTATTTGCACGTAAAACAACGT
P46	AGACAGCAGAAACGAAAGAGGAAATAAATCGAGGTGACAGTTAAAT
P47	CGAGGGTACTTTTTCATGAACGGGGTCATAATGCCGAGCCACCACC
P48	TAAAGCCTCCAGTACCTCATAGTTAGCG
P49	AATATGCAACTACCATCATAGACCGGAACCGC
P50	AGAAATCGTTAGACTACCTTTTTAAGGCGTTCTGACCTTTTTGCA
P51	CTAAATCGGTCAGAATTAGCAAAATTAAGCAATAAAATAATA
P52	AAATCAGCTCATTTTTTAACCATTTTGTTAAAATTCGCATTA
P53	ATAGCGAGAGGCTATCATAACCAAATCCCAAAGAAAATTTCATCCTCAT
P54	GAACTGGCTCATTACAACTTTAATCATTCTTGAGATTACTTA
P55	ACGCGAGAGAAGGCCATGTAATTTAGGCCAGGCTTAATTGAGAATCGC
P56	TAATATCAAAGGCACCGCTTCTGGCACT
P57	TTTCCATGGCACCAACCTACGTCATACA
P58	AAGACAAATCAGCTGCTCATTCAGTCTGACCA
P59	CCGTAATCAGTAGCGACAGAATCTAATTATTCATTAAAAAGG
P60	CTGGCATTAGGAGAATAAAATGAAGAAACGATTTTTTGAGTA
P61	CGCGCCGCCACCAGAACAGAGCCATAAAGGTGGAA
P62	TAGCCCGGAATAGGTGTAAGGATAAGTGCCGTCGA
P63	AAGGCTCCAAAAGGAGCCTTTATATTTTTTCACGTGCTACAGTCACCCT
P64	CAAAATCACCGGAACCAGAGCCAGATTTTGTCACAATCACAC
P65	AATTGTGTCGAAATCCGCGGCACACAACGGAGATTTGTATCA
P66	CCTCGTCTTTCCACCACCGGAACCGCCTCCCTCA
P67	CCGTGTGATAAATAACCTCCGGCTGATG
P68	CCCAGCTACAATGACAGCATTTGAGGCAAGTTGAGAAATGAA
P69	TATTTAAATTGCAGGAAGATTG
P70	AAGGGATATTCATTACCGTAATCTATAGGCT
P71	ACCAGACCGGATTAATTCGAGC
P72	AAGGCCTGTTTAGTATCATGTTAGCTACCTC
P73	AGCAACAAAGTCAGAAATAATATCCAATAATCGGCTCAGGGA
P74	TGAGTAAAGGATAAGTTTAGCTATATCATAGACCATTAGATA

Staple ID	Sequence (5' to 3')
P75	GAGTCTGGATTTGTTATAATTACTACATACACCAC
P76	TTCGGTCCCATCGCATAGTTGCGCCGACATGCTTTCGAGGTG
P77	CGTGTCAAATCACCATCTAGGTAATAGATTT
P78	GGAACCATACAGGCAAGGCAAATCAAAAAGACGTAGTAGCAT
P79	ATTTGGAAGTTTCATGCCTCAACATGTTTTA
P80	AATTTCTTAAACCCGCTTAATTGTATCGTTGCGGGCGATATA
P81	GAGCATTTATCCTGAATCAAACGTGACTCCT
P82	TTATAAGGGTATGGAATAATTCATCAATATA
P83	TAACGACATTTTTACCAGCGCCAAAGAAAGTTACCAGAACCCAAA
P84	AAAGATTACAGAACGGGAGAAGGAAACGTCACCAATGAAACCA
P85	GCTGTAGTTAGAGCTTAATTG
P86	AGTTTCCAACATTATTACATTATAC
P87	GGGATATTGACGTAGCAATAGCTAAGATAGC
P88	AACAAGAGCCTAATGCAGAACGCGC
P89	AGTTTATTGTCCATATAACAGTTGATTC
P90	TATTGAAAGGAATTGAGGTAG
P91	AATAGAAAAAAATAAACGTCTGAGAGGAATATAAGAGCAACACTATGAT
P92	TCGTGCCGGAGTCAATAGTGAATTTGCAGAT
P93	TTAGTTTGAGTGCCCGAGAAATAAAGAAATTGCGTAGAGATA
P94	TTGGTAGAACATTTAATTAAGCAAC
P95	TAACATCCAATAAATGCAAAGGTGGCATCAACATTATGAAAG
P96	TAAGTTTACACTGAGTTTCGT
P97	AGAACTTAGCCTAATTATCCCAAGCCCCCTTATTAGCGTTTGCCA
P98	ACCGCCACCCTCAGAACCCGTACTCTAGGGA
P99	TTAGCCCTGACGAGAAACACCAGAAATTGGGGTGAATTATTTTAA
P100	ATAAAGTCTTTCCTTATCACT
P101	ATTTCCTGATTATCAGATGATGGCTTTAAAAAGACGCTAAAA
P102	ACATAAGTAGAAAAATCAAGAAGCAAAAGAAGATGTCAT
P103	TTCATCGGCATTTTCGGTCATATCAAAA
P104	GAACCGCCACCCTCCATATCATACC
P105	ACTAATGCCACTACGAATAAA
P106	CAAGCCGCCCAATAGCAAGTAAACAGCCATATTATTTTGCCATAAC
P107	TGAAAATCCGGTCAATAACCTAAATTTTAGCCTTT
P108	CCTCGTTTACCAGAAACCAAA
P109	CAAATTATTCATTTCAATTACCTGAGTA
P110	ATTTCAACCAAAAATTCTACTAATAGTTAGTTTCATTTGGGGCGCGAGC
P111	AGGCTTGCGAGACTCCTCAAGAGAAAAGTATTCGGAAC
P112	AATATTCATTGAATCCATGCTGGATAGCGTCCAAT

Staple ID	Sequence (5’ to 3’)
P113	CTAGTCAGTTGGCAAATCAACAGTCTTTAGGTAGATAACAAA
P114	TATGACTTTATACATTTTTTTTTAATGGAAACAGTACACCGT
P115	ACTAAAGAGCAACGTGAAAATCTCCACCCACAACTAAAGGAA
P116	TTGCGAATAATATTTACAGCGGAGTGAGGTAAAATTTTGAGG
P117	CCGACTTGTTGCTAAAATTTATTTAGTTCGCGAGAGTCGTCTTTCCAGA
P118	ATTGTTATCTGAGAAGAAACCAGGCAAAGCGCCATTCGTAGA
P119	AGTACCGCATTCCACAACATGTTCAGCCTTAAGGTAAAGTAATTC
P120	AAACTCACAGGAACGGTACGCCAGTAAAGGGGGTGAGGAACC
P121	CGCTTTCCAGTTAGCTGTTTAAAGAACGT
P122	GGCGAAGCACCGTAATAACGCCAGGGTTTTCCCAGTCATGGG
P123	TTTACCAGTCCCGGCCTGCAGCCCACTACGGGCGCACCAGCT
P124	GGCAACACCAGGGTCTAATGAGTGAGCTCACAACAATAGGGT
P125	GAAGGAGCGGAATTATCATCATATATCATTTACATAGCACAA
P126	CGCGCTACAGAGTAATAAAAGGGACATTCTGATAGAACTTAG
P127	GTAATTAATTTAGAATCTGGGAAGGGCGATCGGTGCGGCAAA
P128	GGATGTGGTTTGCCCCAGCAG
P129	GCCAGCAGTTGGGCGCAAATCAGGTTTCTTGCCCTGCGTGGT
P130	TATCAGCAACCGCAAGAATGCCAATGAGCCTGAGGATCTATC
P131	GAGAACAATATACAAAATCGCGCAGAGGCGATTCGACAAATCCTTTAAC
P132	GTAAAACGACGGCCCATCACCCAAATCAGCGC
P133	ACGGGCCGATAATCCTGAGAAGTGTTTTTATGGAGCTAACCG
P134	TGCTAAATCGGGGAGCCCCCGATTTAGAGCTAGCAGAACATT
P135	ACGCGGTCCGTTTTTGGGTAAGTGA
P136	GCGTCCACTATTCCTGTGTGAAATGCTCACTGCC
P137	CGTACTATGGTAACCACTAGTCTTTAATGCGCGAACTGAATC
P138	AGAATTTTAGAGGAAAACAATATTACCGCCAGCTGCTCATTT
P139	TTGGGCGGCTGATTTCGGCAAAATCCCT
P140	TGGTGGTTGTTCCAGTTTGGAACA
P141	AGTCGCCTGATACTTGCATAACAGAATACGTGGCACAGCTGA
P142	TGCTGATTGCCGTTGTCATAAACATCGGGCGG
P143	TGAGTGTTCCGAAAGCCCTTCACCGCCTAGGCGGTATTA
P144	TGAGCAAATTTATACAGGAATAACATCACTTGCCTGAGTCTT
P145	CCTGCGCTGGGTGGCGAGAAAGGAAGGGAAGGAGCGGGGCCG
P146	CGTACAGGCCCCCTAACCGTCCCCGGGTACCGAGCGTTC
P147	TTTAGATTCACCAGTCACACGACCGGCGCGTGCTTTCCCAGA
P148	CCCCGCTAGGGCAACAGCTGGCGAAAGGGGGATGTGCTTATT
P149	TCACAGCGTACTCCGTGGTGAAGGGATAGCTAAGAGACGAGG
P150	TGCGTGTTCAGGTTGTGTACATCG

Staple ID	Sequence (5’ to 3’)
P151	AGGGAGCCGCCACGGGAACGGATAGGCGAAAGCATCAGCACTCTG
P152	AAGAAAGCGCTGAACCTCAAATATTCTAAAGGAAAGCGTTCA
P153	AGCGCAGCTCCAACCGTAATCATGGTCACGGGAAACCT
P154	CCTCATCACCCCAGCAGGCCTCTTCGCTATTACGCCAGTGCC
P155	GTCGCGTGCCTTCGAATTGTCAAAG
P156	TTCGGGGTTTCTGCCAGGCCTGTGACGATCC
P157	AGAGAAAATCCAGAGAGTTGCAGCAAATC
P158	TGCCATCCCACGCAGGCAGTTCCTCATTGCCGTTTTAAACGA
P159	GCCCGAGTACGAGCCGGAAGC
P160	GAGGCCAAGCTTTGAATACCAAGTACGGATTACCTTTTCAAA
P161	ACGTAAGAATTCGTTCTTAGAAGAACTCAAACTATCGGATAA
P162	TAAAACCGTTAAAGAGTCTGTCCATCCAGAAACCACACAATC
P163	ACGAGCGGCGCGGTCAGGCAAGGCGATTAAGTTGGGTAAAAC
P164	TTTTCCAGCATCAGCGGGGCTAAAGAACCTCGTAGCACGCCA
P165	CAAAGCACTAGATAGCTCCATTCAGGCTGCGCAACTGTCTTG
P166	ATTGCGTTGCTGTTATCCGCTCACAATTCCAAACTCACTTGCGTA
P167	GAGAGATAGACTTTACGGCATCAGA
P168	TGACCGCGCCTTAATTTACAATATTTTTGAATGGCTATCACA
P169	CCTAATTTAACAAACCCTCAATCAATATCTGATTCGCTAATC
P170	TTAACTCGGAATTAGAGTAAATCAATATATGTGAGTGATTCT
P171	ATGAAGGGTAAAGTTCACGGTGCGGCCATGCCGGTCGCCATG
P172	ACATAAAGCCCTTACACTGGTCGGGTTAAATTTGT
P173	AAATGCGGAAACATCGGTTTTCAGGTTTAACGTCAGATTAAC
P174	GTCGCAGAAAAACTTAAATTTGCC
P175	GAATTCGTCTCGTCGCTGGGTCTGCAATCCATTGCAACACGG
P176	GCGAAAATCCCGTAAAAAAAGCCGTGGTGCTCATACCGGCGTCCG
P177	CTTGTAGAACGTCAGCGGCTGATTGCAGAGTTTTTCGACGTT
P178	TCATACATTTAATACCGATAGCCCTAAAACATCGAACGTAAC
P179	TACGGCTGGAGGTGCGCACTCGTCACTGTTTGCTCCCGGCAA
P180	AAATGACGCTAAATGGATTATTTACATTGGCGAATACCTGGA
P181	AACAACAGGAAGCACGTCCTTGCTGGTAATATCCAGAAACGC
P182	TGCATTAATGAGCGGTCCACGCTCACTGCGCCACGTGCCAGC
P183	ACCTGACGGGGAAAGCCGGCGAACCAAGTGTCTGCGCGTTGC
P184	CCAGCCTCCGATCCTCATGCCGGA
P185	GCTGGTCTGGTCAGGAGCCGGAATCCGCCGTGAACAGTGCCA
P186	GCGAATCAGTGAGGCCACCGAGTAGTAGCAACTGAGAGTTGA
P187	GGCCAACGCGCGGGGAGGGCCCTGTGTTTGA
P188	AGCTTTCAGAGGTGGCGATGGCCAGCGGGAAT

Staple ID	Sequence (5’ to 3’)
P189	ATTAGCGGGGTTTTGCTCAGTACCAGGCTGACAACAAGCTG
P190	TGCCCGTATAAACAGTGTGCCTTCTGGTAA
P191	AGAAAACGAGAATGACCATAAATCTACGCCCCTCAAATGCTTTA
P192	ATAACTATATGTAAATGCTTAGGATATAAT
P193	AGGAATCATTACCGCGTTTTTATAAGTACC
P194	GATTAGAGAGTACCTTAACTCCAACAGG
P195	CCTTAAATCAAGATTAGCGGGAGGCTCAAC
P196	GCATGTAGAAACCAATCCATCCTAGTCCTG

Table S5. Biotin-modified staples from the 5’ to the 3’ end for the pillar-shaped DNA origami structure.

Sequence (5’ to 3’)	Function	Replace
**Biotin**-AGAAAACGAGAATGACCATAAATCTACGCCCCTCAAATGCTTTA	Biotin at 5’	P189
**Biotin**-ATTAGCGGGGTTTTGCTCAGTACCAGGCTGACAACAAGCTG	Biotin at 5’	P190
**Biotin**-GCATGTAGAAACCAATCCATCCTAGTCCTG	Biotin at 5’	P191
**Biotin**-GATTAGAGAGTACCTTAACTCCAACAGG	Biotin at 5’	P192
**Biotin**-TGCCCGTATAAACAGTGTGCCTTCTGGTAA	Biotin at 5’	P193
**Biotin**-CCTTAAATCAAGATTAGCGGGAGGCTCAAC	Biotin at 5’	P194
**Biotin**-AGGAATCATTACCGCGTTTTTATAAGTACC	Biotin at 5’	P195
**Biotin**-ATAACTATATGTAAATGCTTAGGATATAAT	Biotin at 5’	P196

Table S6. Staples from the 5’ to the 3’ end for the pillar-shaped DNA origami structure with extensions for pyrene-modified staples binding.

Sequence (5’ to 3’)	Function	Replace
ATATTTCCTCTACCACCTACATCACTAATTAGCGGGGTTTTGCTCAGTACCAGGCTGACAACAAGCTG	External labeling with pyrene	P189
ATATTTCCTCTACCACCTACATCACTAAGAAAACGAGAATGACCATAAATCTACGCCCCTCAAATGCTTTA	External labeling with pyrene	P190
ATATTTCCTCTACCACCTACATCACTAATAACTATATGTAAATGCTTAGGATATAAT	External labeling with pyrene	P191
ATATTTCCTCTACCACCTACATCACTAGCATGTAGAAACCAATCCATCCTAGTCCTG	External labeling with pyrene	P192
ATATTTCCTCTACCACCTACATCACTATGCCCGTATAAACAGTGTGCCTTCTGGTAA	External labeling with pyrene	P193
ATATTTCCTCTACCACCTACATCACTAAGGAATCATTACCGCGTTTTTATAAGTACC	External labeling with pyrene	P194

Sequence (5' to 3')	Function	Replace
ATATTTCCTCTACCACCTACATCACTAGATTAGAGAGT ACCTTAACTCCAACAGG	External labeling with pyrene	P195
ATATTTCCTCTACCACCTACATCACTACCTTAAATCAA GATTAGCGGGAGGCTCAAC	External labeling with pyrene	P196
GTGATGTAGGTGGTAGAGGAAATAT-**pyrene**	Pyrene at 3'	-

Table S7. Staples from the 5' to the 3' end for the pillar-shaped DNA origami structure for Distance determination from fluorescence lifetimes.

Sequence (5' to 3')	Function	Replace
AGACAGCAGAAACGAAAGAGGAAATAAATCGAGGTG ACAGTTAAAT-**ATTO542**	Dye ATTO542 at 3' (11.6 nm)	P46
AATATGCAACTACCATCATAGACCGGAACCGC-**ATTO542**	Dye ATTO542 at 3' (15.9 nm)	P49
CATTTGAGATAACCCACGAAACAATG-**ATTO647N**	Dye ATTO647N at 3' (15.9 nm)	P37
AAGGGATATTCATTACCGTAATCTATAGGCT-**ATTO647N**	Dye ATTO647N at 3' (23.4 nm)	P70
ACGGGCCGATAATCCTGAGAAGTGTTTTTATGGAGCT AACCG-**ATTO647N**	Dye ATTO647N at 3' (52.5 nm)	P133

Table S8. Staples from the 5' to the 3' end for the pillar-shaped DNA origami structure for Expanding FRET (horizontal orientation).

Sequence (5' to 3')	Function	Replace
TGCAATGGGATAGGTCAAAAC	Exchange staple	P1
ATGAAAGGGTGAGAAGGCATC	Exchange staple	P1
AACCGTGTCATTGCAACGGTAATATATTTTAA-**ATTO647N**	Dye ATTO647N at 3' (FRET acceptor)	P2
ACATCTAGCTGGCATAGCCACATTATTCATCGGTCAGA TGATATTCACAAACCAA	Exchange staple	P3/P4
CTGTATGGGATTACCGTTAGTATCA	Exchange staple	P5
CCATAATGCCAGGCTATCAAGGCCGGAG-**ATTO542**	Dye ATTO542 at 3'	P6

Table S9. Staples from the 5' to the 3' end for the pillar-shaped DNA origami structure for Expanding FRET (diagonal orientation).

Sequence (5' to 3')	Function	Replace
TGCAATGGGATAGGTCAAAAC	Exchange staple	P1
ATGAAAGGGTGAGAAGGCATC	Exchange staple	P1
AACCGTGTCATTGCAACGGTAATATATTTTAA-**ATTO647N**	Dye ATTO647N at 3' (FRET acceptor)	P2
AAACTTCAGAAAAGTTTTGAATACCCCAAC	Exchange staple	P7

Sequence (5' to 3')	Function	Replace
ATTCATCAAAAATAATTCGCGTCTTAGCCAGACCCGTC	Exchange staple	P8
CATCCAAAAATAAACGTTAGCCTCAGA	Exchange staple	P9
GGATTGAGTGAGCGAGTAACACTTT	Exchange staple	P10
GCATACCCTGTCGGGAGATAGAACCCTCATCGTA-**ATTO542**	Dye ATTO542 at 3'	P11

Table S10. Staples from the 5' to the 3' end for the pillar-shaped DNA origami structure for Expanding FRET (vertical orientation).

Sequence (5' to 3')	Function	Replace
TGCAATGGGATAGGTCAAAAC	Exchange staple	P1
ATGAAAGGGTGAGAAGGCATC	Exchange staple	P1
AACCGTGTCATTGCAACGGTAATATATTTTAA-**ATTO647N**	Dye ATTO647N at 3' (FRET acceptor)	P2
ATGCTGAGAGTCTACAAGGAGAGGGAACGCCAAAAGGA	Exchange staple	P12
CCCGGTTGATAAAGCATGTCAATCATATTTTTAACAAGAGAATCG-**ATTO542**	Dye ATTO542 at 3'	P13

Table S11. Staples from the 5' to the 3' end for the pillar-shaped DNA origami structure for Graphene biosensing.

Sequence (5' to 3')	Function	Replace
AAGGGATATTCATTACCGTAATCTATAGGCT-**ATTO542**	Colocalization dye ATTO542 at 3' (23.4 nm)	P70
AATATGCAACTACCATCATAGACCGGAACCGCCGCGCCGCCA**TTAAGTGGGATGGACAGACGCGCG**CATCCCACTTAA-**ATTO643**	Capture strand with dye ATTO643 at 3'	P49
AGAAATCGTTAGACTACCTTTTTAAGGCGTTCTGACCTTTTTGCATTTTAAGTGGGATG	Closing strand	P50
TTAGTTTGAGTGCCCGAGAAATAATTTAGCGTATAGCATA	Biasing strand "old"	P93
TTAGTTTGAGTGCCCGAGAAATAATTTAGCGTTTAG	Biasing strand "new"	P93
TATGCTATACGCTATTAAGTGGGATGCGCGCGTCTGTC	Target strand "old"	-
CTAAACGCTATTAAGTGGGATGCGCGCGTCTGTC	Target strand "new"	-

Table S12. Core staples from the 5' to the 3' end for the L-shaped DNA origami structure for Distance determination from fluorescence lifetimes, Dynamics with GET and Expanding FRET. Staples L197-L252 were left out in the experiments with the L-shaped DNA origami structure labeled with 42 pyrene molecules.

Staple ID	Sequence (5' to 3')
L1	ATCCAGAACAATATTAGTCCATCAGGAACGGT

Staple ID	Sequence (5’ to 3’)
L2	CGTGCCTGTTCTTCGCATCCAGCGCCGGGTTA
L3	ATAATCAGAAAAGCCCAACATCCACTGTAATA
L4	CATAGGTCTGAGAGACAAATCGTCGAATTACC
L5	ATTGCCCTTCACCGCCCCAGCTGCTTGCGTTG
L6	TTCGTAATCATGGTCATCCATCAGTTATAAGT
L7	CCCGCCGCGCTTAATGAAAGCCGGCGAACGTG
L8	AGGCGAAAATCCTGTTGTCTATCACCCCCGAT
L9	GCTGCGCAACTGTTGGCAGACCTATTAGAAGG
L10	CTGCAACAGTGCCACGTATCTGGTAGATTAGA
L11	AACAGAGGTGAGGCGGCAGACAATTAAAAGGG
L12	AAATCCCGTAAAAAAACGTTTTTTGGACTTGT
L13	GGCTTAGGTTGGGTTAAGCTAATGATTTTCGA
L14	TATTTTGTTAAAATTCGGGTATATATCAAAAC
L15	GTATAAGCAAATATTTTAGATAAGTAACAACG
L16	CCAGCCAGCTTTCCGGGTAATGGGGTAACAAC
L17	GGGGTCATTGCAGGCGGGAATTGACTAAAATA
L18	TGTTGCCCTGCGGCTGATCAGATGCAGTGTCA
L19	GGAAACCAGGCAAAGCGTACATAAGTGAGTGA
L20	CTCTCACGGAAAAAGAACGGATAAAAACGACG
L21	ATCGGCAAAATCCCTTACGTGGACTCCAACGT
L22	TCAAATCACCATCAATACGCAAGG
L23	GCAGTTGGGCGGTTGTCCAGTTATGGAAGGAG
L24	CTTCTGACCTAAATTTGCAGAGGCCAGAACGCAATTTACG
L25	ATCAAACTTAAATTTCTGGAAGGGCCATATCA
L26	TATCATTTTGCGGAACATCCTGATATAAAGAA
L27	GACCGTGTGATAAATACAAATTCT
L28	TGATTGCTTTGAATACAAACAGAATGTTTGGA
L29	GCCGGGCGCGGTTGCGCCGCTGACCCCTTGTG
L30	GTACTATGGTTGCTTTTTTAGACACGCAAATT
L31	GGGCCTCTTCGCTATTACGTTGTACCTCACCG
L32	GCAGCAAGCGGTCCACAAGTGTTTTGAGGCCA
L33	AACGTTATTAATTTTACAACTAATCAGTTGGC
L34	GAAATTGTTATCCGCTCACATTAAATTAATGA
L35	CCAGCTTACGGCTGGAAACGTGCCCGTCTCGT
L36	GCAGAGGCGAATTATTTTTCATTTGCTATTAA
L37	CATTGCCTGAGAGTCTTTATGACCATAAATCATTTCATTT
L38	CTAGCTGATAAATTAACAGTAGGG

Staple ID	Sequence (5’ to 3’)
L39	AAATCAGCTCATTTTTGTGAGCGAATAGGTCA
L40	TATTTTTGAGAGATCTGCCATATTTCCTCTACTCAATTGA
L41	CAGGAAAAACGCTCATACCAGTAAATTTTTGA
L42	ACAGTTGAGGATCCCCAGATAGAACTGAAAGC
L43	ACGATAAACCTAAAACAAAGAATACACTAAAACATTACCCAACAAAGC
L44	AGAAACAGCTTTAGAAGGAAGAAAAATCTACGATTTTAAGCATATAAC
L45	GCACCCTCCGTCAGGTACGTTAGTAAATGAATAGTTAGCGTCAATCAT
L46	AGTTGATTAGCTGAAAAGAGTACCTTTAATTGTTAATTCGGACCATAA
L47	CTCAAATGTTCAGAAATGGAAGTTTCACGCGCATTACTTCAACTGGCT
L48	TTTCATCGAATAATATCCAGCTACAATACTCCAGCAATTTCTTTACAG
L49	TGCTCATTCTTATGCGTTAATAAAACGAACTATATTCATTGGCTTTTG
L50	GGCACCAAAACCAAAAGTAAGAGCAACACTATAGCAACGTAAATCGCC
L51	AAGGGAACCGGATATTCACTCATCTTTGACCCGTAATGCCATCGGAAC
L52	ATATTCACCGCCAGCATTGACAGGCAAAATCA
L53	CGGAATCTCAGGTCTGTTTTAAATATGCATGCGAACGAATCATTG
L54	AAAGACAAATTAGCAAGTCACCAATGAAACCA
L55	TCGATAGCAGCACCGTAAAATCACGTTTTGCT
L56	TGAATTACCAGTGAATGGAATTACGAGGCATATAGCGAGAGAATCCCC
L57	TAGTTGCCAGTTGCGGGAGGTTTTGAAGATCAATAA
L58	GCCCCCTGGTGTATCACCGTACTC
L59	AATAAGTTAGCAAAAACGCAATAATAACGAGAATTAAAAGCCCAA
L60	CAAAAGAATAAAATACCCAGCGATTATACCAAGCGCGAA
L61	TTTTCATCGGCATATTGACGGCACCACGG
L62	GGGGCGCGCCCAATTCACTAAAGTACGGTGTCACGAGAATAGCTTCAA
L63	CCGGCAAATCGGCGAAGTGGTGAAGGGATAG
L64	ATCAAAAAGTCATAAAACGGAACAACATTATCAACTTTAGTAGAT
L65	TTAGTTTGCCTGTTTAGGTCATTTTTGCGGATAGGAAGCCGACTATTA
L66	GCGAGAAAAGGGATGACGAGCACGTATAACGTGCTTTTCACGCTGAAGAAAGC
L67	CCCTGAACAAATAAGAAACGCGAGGCGTT
L68	CTGAGGCCAACGGCTACAGAGGTTTCCATT
L69	ACATTCTGAAGAGTCTCCGCCAGCAGCTCGAA
L70	AAATCAACACGTGGCATCAGTATTCTCAATCC
L71	TTATACTTAGCACTAAAAAGTTTGTGCCGCCA
L72	CCAACATGACGCTCAATGCCGGAGGAAATACC
L73	CCGGAACCGCAAGAAAGCAATAGCTATCTTACTCACAATCCGATTGAG
L74	GTAAGAATAGTTGAAACTTTCGCAAACACCGC
L75	GCCAGTGCGATTGACCCACCGCTTCTGGTGCC

Staple ID	Sequence (5’ to 3’)
L76	AGGAAACCGAGGACGTAGAAAAAGTACCG
L77	CTGCGCGGCTAACTCACAATTCCACACAACATACGAGTACCGGGGCTCTGTGGGTG TTCAG
L78	AATTACATAGATTTTCAATAACGGATTCGCC
L79	ATAACCTTATCAACAAAAATTGTATAACCTCC
L80	CCAGAATGGAGCCGCCAATCAAGTTTGCC
L81	TTTTTTAATGCACGTACAAGTTACCCATTCAG
L82	CATTATACGGTTTACCCATAACCCTCGAAATACAATGTTTAAACAGGG
L83	CTTTTGCGTTATTTCAATGATATTCAACCGTT
L84	GACAGATGGACCTTCATCAAGAGCCCTGAC
L85	ACAAGAAATAGGAATCCCAATAGCAAGCAAATATAGCAGCATCCTGAA
L86	AAATTATTTGGAAACAGCCATTCGAAAATCGC
L87	CACTCATGAAACCACCTTAAATCAAGATTGAGCGTCTTTTTGTTT
L88	GCCTAATTATCATATGATAAGAGATTTAGTTAATTTCAT
L89	GAGGGTAGTTGCAGGGTGCTAAACAACTTTCACGCCTGGAAAGAG
L90	AGAGCCGCAAACAAATGAGACTCCTCAAGAGATTAGCGGGCAGTAGCA
L91	ATTGCGTTTAACAACATTTCAATTACCTGAGCAAAAGGGAGAAACAGGTTTAAGAT GATGG
L92	CCACCCTCTGTTAGGAAGGATCGTCTTTCCAGCAGACGATTATCAGCT
L93	GCCAGTACGTTATAAGGCGTTAAATAAGAATAAACACAAAT
L94	CAATTCATATAGATAATAAATCCTTTGCCCG
L95	GCCGTCACAATATAAAAGAAACCACCAGAAGGAGCGGACTCGTATTACATTTGTCA AATAT
L96	TACCAGTAACGCTAACAGTTGCTATTTTGCACCCCATCCT
L97	GTCGAAATCCGCGACCTGCTCCACCAACTTTTAGCATTC
L98	GTCCACTAAACGCGCGGACGGGCAACAGCTG
L99	AACCGTTTCACACGGGAAATACCTACATTTTGACGCTAAACTATCACTTCTTTAACA GGAG
L100	CGCTGGCACCACGGGAGACGCAGAAACAGCGG
L101	CAAATCGTCAGCGTGGTGCCATCCCACGCAA
L102	GCCGATTAAGGAAGGGCGCGTAACCACCACA
L103	TGTAGCTCAACATTTACCCTCGAAAGAC
L104	GAGAAACATTTAATTTTACAGGTAGAAAG
L105	TTGAGTAAGCCACCCTCAGAACCG
L106	TTAGAGCTATCCTGAGGCTGGTTTCAGGGCGC
L107	TTCACCAGGTAGCAATGGCCTTGCTGGTAAT
L108	CGCTCACTATCAGACGGTCCGTGAGCCTCCTC
L109	ATTCATATCAGTGATTTGGCATCAGGACGTTGTAACATAAACCAGACG
L110	GGAGGGAAGAGCCAGCAATCAGTAGCGACAGACCAGAACCGCCTC

Staple ID	Sequence (5' to 3')
L111	AACGTCAATAGACGGGGAATACCCAAAAGAACAAGACTCCGTTTTTAT
L112	TGTACTGGTAATAAGTTCAGTGCC
L113	TTCAAATTTTTAGAAAAAACAGGAGCAAACAAGAGAATCGATGAAGGGTGAGATA TTTTA
L114	TAATAAGAAGAGCCACCCTTATTAGCGTTTGCCATTCAACAATAGAAA
L115	TTCTGAAACATGAAAGTGCCGGCCATTTG
L116	CAAACCCTTTAGTCTTACCAGCAGAAGATAA
L117	AAACGGGGTTTTGCTACATAACGCCAAAAAAGGCTTGTAATCTTG
L118	TGGAGCCGGCCTCCGGGTACATCGACATAAAA
L119	CCGAGTAAGCCAACAGGGGTACCGCATTGCAA
L120	ACAAGAACCGAACTGATGTTACTTAGCCGGAAAAGACAGCACTACGAA
L121	AGAACGTTAACGGCGTAATGGGTAAAGGTTTCTTTGCGTCGGTGGTGCTGGTCTTG CCGTT
L122	GGAGCCTTCACCCTCAGAGCCACC
L123	CCCCCTGCGCCCGCTTTAGCTGTTTCCTGTGT
L124	TGCGGGATAGCAGCGACGAGGCGCAGAGAAACGGCCGCGGTAACGATC
L125	TAATAGTATTCTCCGTGCATTAAATTTTTGTT
L126	CACATCCTCAGCGGTGGTATGAGCCGGGTCAC
L127	CACAGACATTTCAGGGATCTCCAAAAAAAAGGTTCTTAAAGCCGCTTT
L128	CCATTACCAAGGGCGACATCTTTTCATAGGCAGAAAGAATAGGTTGAG
L129	ATGAGTGACCTGTGCAGTTTCTGCCAGCACG
L130	AAGCGCATAAATGAAACAGATATAGAAGGCTTAGCAAGCCTTATTACG
L131	ATAAAAATATCGCGTTCTCCTTTTGATAAGAGCTATAT
L132	ATCGGCCTTAAAGAATAAATCAAAAGAATAGCCCGAGACCAGTGAGGGAGAGGGG TGCCTA
L133	CCTGCAGCCATAACGGGGTGTCCAGCATCAGC
L134	ATGGCTACAATCAACTGAGAGCCAGCAGCAAATGAAAAACGAACCTAATGCGCTT GGCAGA
L135	TACAGGCATTAAATTAACCAATAGGAACGCCATCAAAGTCAATCAGAATTAGCCTA AATCG
L136	CCGTCGGAGTAGCATTCAAAAACAGGAAGATT
L137	GTTTTCCCGTAGATGGCAGGAAGATCGCACT
L138	GCCTGTTTGCTTCTGTTACCTTTTAACGTTAA
L139	AAACGGCGCAAGCTTTGAAGGGCGATCGGTGC
L140	TACCGATAGTTGCGCTTTTTCA
L141	CAGTACCATTAGTACCCAGTGCCCGTATAAATTGATGAATTAAAG
L142	CAACTAATGCAGACAGAGGGGCAATACTG
L143	ACCCTCATGCCCTCATTTTCTGTATGGGATTTAGTTAAAGCAGCTTGA
L144	ATAAACAATCCCTTAGTGAATTTATCAAAAT

Staple ID	Sequence (5' to 3')
L145	CCTCAGAGCACAAGAAGAAAAGTAAGCAG
L146	CAGTATGTTTATTTTGCGAAGCCCTTTTTAATTGAGTTCTGAACA
L147	CGGGAAACGAAAAACCTGATGGTGGTTCCGAA
L148	CTTAATTGAGACCGGAAACAGGTCAGGATTAGAGGTGGCA
L149	TCATCAACAAGGCAAATATGTACCCCGGTTG
L150	TGCTTTCGAGGTGAATCTCCAAAA
L151	AGCATGTACGAGAACAATCCGGTATTCTAAGAACGATTTTCCAGA
L152	TCTTACCATAAAGCCATAATTTAGAATGGTTTAGGGTAGC
L153	CGTTGAAAATAGCAAGCCCAATA
L154	GTTGTACCACCCTCATAAAGGCCGGAGACAG
L155	GAAACAACGCGGTCGCCGCACAGGCGGCCTTTAGTGACTTTCTCCACGTACAGACG CCAGG
L156	CAAAGGGCCTGTCGTGTGGCCCTGAGAGAGTT
L157	TTAATTTCATGTTCTATAACTATATGTAAATGCTGATGTCAATAGAATCCTTGACAA AATT
L158	AGCGAACCAGAAGCCTGGAGAATCACAAAGGCTATCAGGT
L159	CGTTGGTAGTCACGACGCCAGCTGGCGAAAGGGGGATATCGGCCTGCGCATCGGCC AGCTT
L160	GGAACCCAAAACTACAAACAGTTTCAGCG
L161	AGGAGGTGGCGGATAAGTATTAAGAGGCTAAATCCTCTACAGGAG
L162	GGAATTAGGTAAATTTTCGGTCATAGCCCCACCGGAACCACCACC
L163	TCTTTAGGCTGAATAATGCTCATTAGTAACAT
L164	TGCGAATAATAATCGACAATGTTCGGTCG
L165	ACGCCAGATGACGGGGCGCCGCTAGCCCCAGC
L166	TAAAGTTTAGAACCGCTAATTGTATCGCGGGGTTTAAGTTTGGCCTTG
L167	ATTATAGCGTCGTAATAGTAAAATGTTTTTT
L168	TTTTTTTTTTTTAAAACTAG
L169	TTTTTGCCTGAGTAGAAGAA
L170	TTTTGATTAAGACGCTGAGA
L171	TTTTGGCGCATAGGCTGGCTAACGGTGTTAAATTGT
L172	TTTGCGTATTGGGCGCTTTT
L173	TAGTCAGAAGCAAAGCGGATTTT
L174	TTTTCGCAAATGGTCAATAAACCATTAGATGC
L175	TTTTTTGCATCAAAAGCCTGAGTAATTTT
L176	TTTTCCATATTATTTATCCCAATCCAAAGTCAGAGA
L177	GAAAGGAGCGGGCGCTAGGTTTT
L178	ATATATATAAAGCGACGACATCGGCTGTCTTTCCTTATCATTTTT
L179	TCAGCAGCAACCGCAATTTT

Staple ID	Sequence (5' to 3')
L180	TTTTGTTTCGTCACCAGTACTGTACCGTAAT
L181	TTTTCTTTACAAACAATTCG
L182	TTTTACCGTTCCAGTAAGCGTCATACATGGCTTCAGTTAAT
L183	TTTTGGAATTTGTGAGAGAT
L184	AGAGCAAATCCTGTCCAGATACCGACAAAAGGTAATTTT
L185	ATACGCAAAGAAAATTATTCATTAAAGGTGAATTTT
L186	TTAATTAAACCATACATACATAAAGGTGGCAATTTT
L187	CTGATAGCCCTAAAACTTTT
L188	TTTTATTGGGCTTGAGATGGCCAGAACGATT
L189	CAGATGAATATACAGTTTTT
L190	TTTTCGGGCCGTTTTCACGG
L191	CCGTGCATCTGCCAGTTTTT
L192	TTTTGCTAATATCAGAGAGATAACCCCGCCACCGCG
L193	ACAAAGTATGAGGAAGCTTTGAGGACTAAAGATTTT
L194	TTTCGACTTGATCGAGAGGGTTGATATAAGTATTTT
L195	TTTTCCCTCAGAGCCACCACCCTCAGAAAGCGCTTA
L196	GAGCCGATATAACAACAACCATCGCCCTTTTTTT
L197	CCGAATCTAAAGCATCTTTT
L198	TTTTACCTTGCTGAACCAGG
L199	AGTGTGCTGCAAGGCGTTTT
L200	TTCCGGAATCATAATTTTTT
L201	CATAATAATTCGCGTCTTTT
L202	TTTTAGAGCGGGAGCTAGAT
L203	TTTTGGAACCTAAGTCTCTGAATTTTTTTTTT
L204	AATGCAATAGATTAAGGGCTTAGAGCTTATTTT
L205	TTTTACTGTAGCCTCAGAACCGCCATTTT
L206	TTTTCAGGGTGGTTTTTCTT
L207	TTTTCATATAAAAGAAAGCCGAACATTTT
L208	CATGTTTACCAGTCCCTTTT
L209	TTTTAAACATCAAGAAAAAA
L210	TTTTAACAGTACCTTTTACA
L211	ATTTAGAAGTATTAGATTTT
L212	TTTTAGAACGCGAGAAAACTTT
L213	TTTTATTGCTGAATATAATACATTTTTTT
L214	TTTTTTAGGAATACCACAGTAGTAATTTT
L215	TTTTGTGTAAAGCCTGGCGG
L216	TTTTATCGCCATTAAAAATA

Staple ID	Sequence (5’ to 3’)
L217	TTTTGAACAACTAAAGGAACACTGATTTT
L218	TTTTTTATCACCGTCACAGCGTCAGTTTT
L219	TTTTTGGCCTTCCTGTATAA
L220	TTTTTCCAAGAACGGGTGCGAACCTTTTT
L221	TTTTTGGATTATTTACAGAA
L222	TCACCGGAAGCATAAATTTT
L223	TTTTCTTTTTCACAACGGAGATTTGTTTT
L224	ACAAATTATCATCATATTTT
L225	TTTTACGCATAATGAGAATAGAAAGTTTT
L226	TTTTGCCTCAGAGCATAAAGAAAATTAAGCAATAAATTTT
L227	TAGTAATAACATCACTTTTT
L228	TTTTCCCTTACACTGGTTGC
L229	AGATGAAGGGTAAAGTTTTT
L230	TTTTCCCGACTTACAAAATAAACAGTTTT
L231	TTTTTAAACGATGCTGATGG
L232	ACATAGCGATAGCTTATTTT
L233	TTTTATTAAGTTGGGTACGC
L234	TTTTTTGTTCCAGTTTGGAACAAGA
L235	TCGAAGATGATGAAACTTTT
L236	TTTTTATCATCGCCTGAACAGACCATTTT
L237	ACCTCGTCATAAACATTTTT
L238	TTTTGCGCTGGCAAGTGTAG
L239	TTTTTTGAGGGGACGACGAC
L240	CTCCAATCGTCTGAAATTTT
L241	AAAACGGTAATCGTTTTTTT
L242	TTTTTTCCTGATTATCACGT
L243	TTTTTAGACTGGCATCAGTTGAGATTTTTT
L244	CGGCCTCGTTAGAATCTTTT
L245	TTTTTAGCCCGGAATAGCCTATTTCTTTT
L246	TTTTGTGTAGGTAAAGATTC
L247	TTCATAGGGTTGAGTGTTTT
L248	TTTTGAATGCCAACGGCAGC
L249	TTTTACTAGAAAAAGCCTGTT
L250	TTTTAAGTTACCAGGGTAATTGAGCTTTT
L251	TGCGGCCAGAATGCGGTTTT
L252	TTTTAGTAATTCAATCGCAAGACAATTTT

Table S13. Biotin-modified staples from the 5' to the 3' end for the L-shaped DNA origami structure.

Sequence (5' to 3')	Function	Replace
Biotin-ATCCAGAACAATATTAGTCCATCAGGAACGGT	Biotin at 5'	L1
Biotin-CGTGCCTGTTCTTCGCATCCAGCGCCGGGTTA	Biotin at 5'	L2
Biotin-ATAATCAGAAAAGCCCAACATCCACTGTAATA	Biotin at 5'	L3
Biotin-CATAGGTCTGAGAGACAAATCGTCGAATTACC	Biotin at 5'	L4

Table S14. Staples from the 5' to the 3' end for the L-shaped DNA origami structure with extensions for pyrene-modified staple binding. Staples marked with the numbers 1-6 or 1-8 were used in the measurements with only 6 or 8 pyrene molecules, respectively (Distance determination from fluorescence lifetimes). In all other measurements, 42 staples were used for external labeling with pyrene molecules.

Sequence (5' to 3')	Function	Replace
ATATTTCCTCTACCACCTACATCACTAATCCAGAACAA TATTAGTCCATCAGGAACGGT	External labeling with pyrene (1)	L1
ATATTTCCTCTACCACCTACATCACTACGTGCCTGTTC TTCGCATCCAGCGCCGGGTTA	External labeling with pyrene (2)	L2
ATATTTCCTCTACCACCTACATCACTAATAATCAGAAA AGCCCAACATCCACTGTAATA	External labeling with pyrene (3)	L3
ATATTTCCTCTACCACCTACATCACTACATAGGTCTGA GAGACAAATCGTCGAATTACC	External labeling with pyrene (4)	L4
ATATTTCCTCTACCACCTACATCACTAATTGCCCTTCA CCGCCCCAGCTGCTTGCGTTG	External labeling with pyrene	L5
ATATTTCCTCTACCACCTACATCACTATTCGTAATCAT GGTCATCCATCAGTTATAAGT	External labeling with pyrene	L6
ATATTTCCTCTACCACCTACATCACTACCCGCCGCGCT TAATGAAAGCCGGCGAACGTG	External labeling with pyrene	L7
ATATTTCCTCTACCACCTACATCACTAAGGCGAAAATC CTGTTGTCTATCACCCCCGAT	External labeling with pyrene	L8
ATATTTCCTCTACCACCTACATCACTAGCTGCGCAACT GTTGGCAGACCTATTAGAAGG	External labeling with pyrene	L9
ATATTTCCTCTACCACCTACATCACTACTGCAACAGTG CCACGTATCTGGTAGATTAGA	External labeling with pyrene (5)	L10
ATATTTCCTCTACCACCTACATCACTAAACAGAGGTGA GGCGGCAGACAATTAAAAGGG	External labeling with pyrene	L11
ATATTTCCTCTACCACCTACATCACTAAAATCCCGTAA AAAAACGTTTTTTGGACTTGT	External labeling with pyrene	L12
ATATTTCCTCTACCACCTACATCACTAGGCTTAGGTTG GGTTAAGCTAATGATTTTCGA	External labeling with pyrene	L13
ATATTTCCTCTACCACCTACATCACTATATTTTGTTAA AATTCGGGTATATATCAAAAC	External labeling with pyrene	L14

Sequence (5' to 3')	Function	Replace
ATATTTCCTCTACCACCTACATCACTAGTATAAGCAAATATTTTAGATAAGTAACAACG	External labeling with pyrene	L15
ATATTTCCTCTACCACCTACATCACTACCAGCCAGCTTTCCGGGTAATGGGGTAACAAC	External labeling with pyrene	L16
ATATTTCCTCTACCACCTACATCACTAGGGGTCATTGCAGGCGGGAATTGACTAAAATA	External labeling with pyrene	L17
ATATTTCCTCTACCACCTACATCACTATGTTGCCCTGCGGCTGATCAGATGCAGTGTCA	External labeling with pyrene	L18
ATATTTCCTCTACCACCTACATCACTAGGAAACCAGGCAAAGCGTACATAAGTGAGTGA	External labeling with pyrene	L19
ATATTTCCTCTACCACCTACATCACTACTCTCACGGAAAAAGAACGGATAAAAACGACG	External labeling with pyrene	L20
ATATTTCCTCTACCACCTACATCACTAATCGGCAAAATCCCTTACGTGGACTCCAACGT	External labeling with pyrene	L21
ATATTTCCTCTACCACCTACATCACTATCAAATCACCATCAATACGCAAGG	External labeling with pyrene	L22
ATATTTCCTCTACCACCTACATCACTAGCAGTTGGGCGGTTGTCCAGTTATGGAAGGAG	External labeling with pyrene	L23
ATATTTCCTCTACCACCTACATCACTACTTCTGACCTAAATTTGCAGAGGCCAGAACGCAATTTACG	External labeling with pyrene	L24
ATATTTCCTCTACCACCTACATCACTAATCAAACTTAAATTTCTGGAAGGGCCATATCA	External labeling with pyrene	L25
ATATTTCCTCTACCACCTACATCACTATATCATTTTGCGGAACATCCTGATATAAAGAA	External labeling with pyrene	L26
ATATTTCCTCTACCACCTACATCACTAGACCGTGTGATAAATACAAATTCT	External labeling with pyrene	L27
ATATTTCCTCTACCACCTACATCACTATGATTGCTTTGAATACAAACAGAATGTTTGGA	External labeling with pyrene	L28
ATATTTCCTCTACCACCTACATCACTAGCCGGGCGCGGTTGCGCCGCTGACCCCTTGTG	External labeling with pyrene	L29
ATATTTCCTCTACCACCTACATCACTAGTACTATGGTTGCTTTTTTAGACACGCAAATT	External labeling with pyrene	L30
ATATTTCCTCTACCACCTACATCACTAGGGCCTCTTCGCTATTACGTTGTACCTCACCG	External labeling with pyrene (6)	L31
ATATTTCCTCTACCACCTACATCACTAGCAGCAAGCGGTCCACAAGTGTTTTGAGGCCA	External labeling with pyrene (7)	L32
ATATTTCCTCTACCACCTACATCACTAAACGTTATTAATTTTACAACTAATCAGTTGGC	External labeling with pyrene	L33
ATATTTCCTCTACCACCTACATCACTAGAAATTGTTATCCGCTCACATTAAATTAATGA	External labeling with pyrene	L34
ATATTTCCTCTACCACCTACATCACTACCAGCTTACGGCTGGAAACGTGCCCGTCTCGT	External labeling with pyrene	L35
ATATTTCCTCTACCACCTACATCACTAGCAGAGGCGAATTATTTTTCATTTGCTATTAA	External labeling with pyrene	L36
ATATTTCCTCTACCACCTACATCACTACATTGCCTGAGAGTCTTTATGACCATAAATCATTTCATTT	External labeling with pyrene	L37

Sequence (5' to 3')	Function	Replace
ATATTTCCTCTACCACCTACATCACTACTAGCTGATAA ATTAACAGTAGGG	External labeling with pyrene	L38
ATATTTCCTCTACCACCTACATCACTAAAATCAGCTCA TTTTTGTGAGCGAATAGGTCA	External labeling with pyrene	L39
ATATTTCCTCTACCACCTACATCACTATATTTTTGAGA GATCTGCCATATTTCCTCTACTCAATTGA	External labeling with pyrene (8)	L40
ATATTTCCTCTACCACCTACATCACTACAGGAAAAACG CTCATACCAGTAAATTTTTGA	External labeling with pyrene	L41
ATATTTCCTCTACCACCTACATCACTAACAGTTGAGGA TCCCCAGATAGAACTGAAAGC	External labeling with pyrene	L42
GTGATGTAGGTGGTAGAGGAAATAT-**pyrene**	Pyrene at 3'	-

Table S15. Staples from the 5' to the 3' end for the L-shaped DNA origami structure for Distance determination from fluorescence lifetimes.

Sequence (5' to 3')	Function	Replace
**ATTO542**-AATAAGTTAGCAAAAACGCAATAATAACGAGAATTAA AAGCCCAA	ATTO542 at 5' at the height of 18.9 nm	L59
**ATTO643**-GAGAAACATTTAATTTTACAGGTAGAAAG	ATTO643 at 5' at the height of 18.9 nm	L104

Table S16. Staples from the 5' to the 3' end for the L-shaped DNA origami structure for Dynamics with GET.

Sequence (5' to 3')	Function	Replace
GGCACCAAAACCAAAAGTAAGAGCAACACTATAGCA ACGTAAATCGCCTTTTTTTTTCGGGCATTTA- **Cy3B**	Pointer-Cy3B at 3'	L43
AGAAACAGCTTTAGAAGGAAGAAAAATCTACGATTTT AAGCATATAACTTTTAAAT	Lower binding site 5nt	L44
GCACCCTCCGTCAGGTACGTTAGTAAATGAATAGTTA GCGTCAATCATTTTTAAAT	Upper binding site 5nt	L45
AGAAACAGCTTTAGAAGGAAGAAAAATCTACGATTTT AAGCATATAACTTTTAAATG	Lower binding site 6nt	L44
GCACCCTCCGTCAGGTACGTTAGTAAATGAATAGTTA GCGTCAATCATTTTTAAATG	Upper binding site 6nt	L45
AGAAACAGCTTTAGAAGGAAGAAAAATCTACGATTTT AAGCATATAACTTTTAAATGC	Lower binding site 7nt	L44
GCACCCTCCGTCAGGTACGTTAGTAAATGAATAGTTA GCGTCAATCATTTTTAAATGC	Upper binding site 7nt	L45
AGAAACAGCTTTAGAAGGAAGAAAAATCTACGATTTT AAGCATATAACTTTTAAATGCC	Lower binding site 8nt	L44
GCACCCTCCGTCAGGTACGTTAGTAAATGAATAGTTA GCGTCAATCATTTTTAAATGCC	Upper binding site 8nt	L45

Table S17. Staples from the 5' to the 3' end for the L-shaped DNA origami structure for Dynamics with GET – tether.

Sequence (5' to 3')	Function	Replace
AATCTACGATTTTAAGAACTGGCTTTTTTTGATCGATC GGATCGATCAAGATCGATCGATGCTATAAACGTCCTTT	Tether	L44
AATCTACGATTTTAAGAACTGGCTTTTTTTGATCGATC GGATCGATCAAGATCGATCGATGCTATAAACGTCCTTT -**Biotin**	Tether Biotin at 3'	L44
CATATAACAGTTGATTAGCT	Exchange staple	L46
GAAAAGAGTACCTTTAATTGTTAATTCGGACCATAA	Exchange staple	L46
CTCAAATGTTCAGAAATGGAAGTTTCACGCGCATTACT TC	Exchange staple	L47
CAATTTCTTTACAGAGAAACAGCTTTAGAAGGAAGAA A	Exchange staple	L48
TTTCATCGAATAATATCCAGCTACAATACTCCAG	Exchange staple	L48
AGGACGT-**Cy3B**-TATAGCATCGATCGATCTTGATCGATCCGATCGATC	Tether complementary Cy3B internally labeled at a T-base	-

Table S18. Staples from the 5' to the 3' end for the L-shaped DNA origami structure for dynamic FRET.

Sequence (5' to 3')	Function	Replace
GGCACCAAAACCAAAAGTAAGAGCAACACTATAGCA ACGTAAATCGCCTTTTTTTTTCGGGCATTTA-**ATTO542**	Pointer-ATTO542 at 3'	L43
TGCTCATTCT-**ATTO647N**-ATGCGTTAATAAAACGAACTATATTCATTGGCTTTTG	Acceptor-ATTO647N-internal	L49
AGAAACAGCTTTAGAAGGAAGAAAAATCTACGATTTT AAGCATATAACTTTTAAATGC	Lower binding site 7nt	L44
GCACCCTCCGTCAGGTACGTTAGTAAATGAATAGTTA GCGTCAATCATTTTTAAATGC	Upper binding site 7nt	L45

Table S19. Staples from the 5' to the 3' end for the L-shaped DNA origami structure for GET tracking.

Sequence (5' to 3')	Function	Replace
GGCACCAAAACCAAAAGTAAGAGCAACACTATAGCA ACGTAAATCGCCTTTTTTTTTCGGGCATTTA-**Cy3B**	Pointer-Cy3B at 3'	L43
AACGAATCATTGTGAATTACCTTTTTTAAATGCC	Lower binding site	L49
GGCACCAAAACCAAAAGTAAGAGCAACACTATAGCA ACTTTTAAATGC	Middle binding site	L50
AGCGTCAATCATAAGGGAACCGGTTTTAAATGCC	Upper binding site	L51
GCACCCTCCGTCAGGTACGTTAGTAAATGAATAGTT	Exchange staple	L45
TGCTCATTCAGTGAATGGAATTACGAGGCATATAGCG AGAGAATCCCC	Exchange staple	L49

Sequence (5’ to 3’)	Function	Replace
ATATTCACTCATCTTTGACCCGTAATGCCATCGGAAC	Exchange staple	L51
ATATTCACCGCCAGCATCGATAGCAGCACCGTAAAAT CACGTTTTGCT	Exchange staple	L52
CGGAATCTCAGGTCTGTTTTAAATATGCATGCG	Exchange staple	L53
GTAAATCGCCAAAGACAAATTA	Exchange staple	L54
GCAAGTCACCAATGAAACCATTGACAGGCAAAATCA	Exchange staple	L55
ATGCGTTAATAAAACGAACTATATTCATTGGCTTTTG	Exchange staple	L56

6. References

[1] L. M. Hall, M. Gerowska, T. Brown, *Nucleic Acids Res.* **2012**, *40*, e108.

[2] A. Gust, A. Zander, A. Gietl, P. Holzmeister, S. Schulz, B. Lalkens, P. Tinnefeld, D. Grohmann, *Molecules* **2014**, *19*, 15824.

[3] J. J. Schmied, R. Dijkstra, M. B. Scheible, G. M. R. De Luca, J. J. Sieber, available at https://www.leica-microsystems.com/science-lab/measuring-the-3d-sted-psf-with-a-new-type-of-fluorescent-beads/, **2016**.

[4] P. C. Nickels, B. Wünsch, P. Holzmeister, W. Bae, L. M. Kneer, D. Grohmann, P. Tinnefeld, T. Liedl, *Science* **2016**, *354*, 305.

[5] Y. Ke, S. M. Douglas, M. Liu, J. Sharma, A. Cheng, A. Leung, Y. Liu, W. M. Shih, H. Yan, *J. Am. Chem. Soc.* **2009**, *131*, 15903.

[6] S. Fischer, C. Hartl, K. Frank, J. O. Rädler, T. Liedl, B. Nickel, *Nano Lett.* **2016**, *16*, 4282.

[7] I. Kaminska, J. Bohlen, S. Rocchetti, F. Selbach, G. P. Acuna, P. Tinnefeld, *Nano Lett.* **2019**, *19*, 4257.

[8] M. Raab, I. Jusuk, J. Molle, E. Buhr, B. Bodermann, D. Bergmann, H. Bosse, P. Tinnefeld, *Sci. Rep.* **2018**, *8*, 1780.

[9] S. Krause, E. Ploetz, J. Bohlen, P. Schuler, R. Yaadav, F. Selbach, F. Steiner, I. Kamińska, P. Tinnefeld, *ACS Nano,* in revision.

[10] X. Li, Y. Zhu, W. Cai, M. Borysiak, B. Han, D. Chen, R. D. Piner, L. Colombo, R. S. Ruoff, *Nano Lett.* **2009**, *9*, 4359.

[11] A. Edelstein, N. Amodaj, K. Hoover, R. Vale, N. Stuurman, *Curr. Protoc. Mol. Biol.* **2010**, *92*, 14.20.1.

[12] J. J. Schmied, C. Forthmann, E. Pibiri, B. Lalkens, P. Nickels, T. Liedl, P. Tinnefeld, *Nano Lett.* **2013**, *13*, 781.

[13] T. Cordes, J. Vogelsang, P. Tinnefeld, *J. Am. Chem. Soc.* **2009**, *131*, 5018.

[14] C. E. Aitken, R. A. Marshall, J. D. Puglisi, *Biophys. J.* **2008**, *94*, 1826.

[15] T. A. Laurence, S. Fore, T. Huser, *Opt. Lett.* **2006**, *31*, 829.

[16] B. Spokoyny, “Photon arrival time correlation,” can be found under https://www.mathworks.com/matlabcentral/fileexchange/64605-photon-arrival-time-correlation, **2017**.

[17] G. L. Lukacs, P. Haggie, O. Seksek, D. Lechardeur, N. Freedman, A. S. Verkman, *J. Biol. Chem.* **2000**, *275*, 1625.

[18] K. A. Velizhanin, T. V. Shahbazyan, *Phys. Rev. B - Condens. Matter Mater. Phys.* **2012**, *86*, 245432.

[19] T. Bian, R. Chang, P. T. Leung, *Plasmonics* **2016**, *11*, 1239.

[20] V. D. Karanikolas, C. A. Marocico, A. L. Bradley, *Phys. Rev. B - Condens. Matter Mater. Phys.* **2015**, *91*, 125422.

[21] S. A. Biehs, G. S. Agarwal, *Appl. Phys. Lett.* **2013**, *103*, 243112.

[22] J. Schnitzbauer, M. T. Strauss, T. Schlichthaerle, F. Schueder, R. Jungmann, *Nat. Protoc.* **2017**, *12*, 1198.
